# Supplementary material for: Genetic control of RNA splicing and its distinct role in complex trait variation
Source: Nat Genet. 2022 Aug 18;54(9):1355–63. doi: 10.1038/s41588-022-01154-4 (PMC9470536; doi:10.1038/s41588-022-01154-4)
Supplement: Supplementary file 1 — Supplementary Figs. 1–24, Tables 1–7 and Note. [file 41588_2022_1154_MOESM1_ESM.pdf]

nature portfolio

https://doi.org/10.1038/s41588-022-01154-4

Supplementary information

Genetic control of RNA splicing and its distinct role in complex trait variation

In the format provided by the authors and unedited

Genetic control of RNA splicing and its distinct role in complex trait variation

Qi *et al.*

Supplementary Note (sections 1-10)

Supplementary Figures 1-24

Supplementary Tables 1-7

Supplementary References

Supplementary Note

1. Transcript-level vs. event-level sQTL mapping strategy

The transcript-level strategies typically involve quantifying isoform expression by counting RNA-seq reads aligned to transcripts, followed by generating splicing phenotypes (e.g., isoform ratios, ratios of mRNA abundance of each isoform to the overall mRNA abundance of the corresponding gene) and testing for associations between genetic variants and the splicing phenotypes[1-3]. They can detect sQTLs arising from complex splicing events because of using annotated transcript isoforms. On the other hand, the event-level strategies typically involve quantifying splicing events by counting RNA-seq reads aligned to specific exons or splice junctions, followed by generating splicing phenotypes (e.g., intron excision ratios)[4-7], which are subsequently used as input into a molecular QTL mapping tool for association tests to detect sQTLs. Compared to the transcript-level strategies, the event-level strategies have the advantage of detecting novel splicing events because of not relying on transcript annotations and being more sensitive to subtle splicing events (i.e., the formations of RNA transcripts by removing or including a small number of nucleotides) because of their specific focus on local splicing events.

2. Simulation study

We calibrated THISTLE and compared it with sQTLseekeR using simulated data. We simulated genotype data of 1,000 unlinked SNPs in 500 individuals using a binomial distribution using the R function rbinom(n, 2, p), with n being the sample size and p being the minor allele frequency (MAF) of the SNP which was generated from a uniform distribution using the R function runif(1, 0.05, 0.5). Assuming a gene with three transcript isoforms, we randomly sampled a SNP as the causal variant with its effect on three isoforms denoted by $\mathbf{b} = \{b_1, b_2, b_3\}$, and an sQTL effect was realised by creating differences between b_1, b_2 and b_3. We generated $\mathbf{b}$ under four different scenarios: 1) $\mathbf{b} = \{0, 0, 0\}$, where the causal variant is neither an sQTL nor an eQTL, 2) $\mathbf{b} = \{2, 2, 2\}$, where the causal variant is an eQTL but not an sQTL, 3) $\mathbf{b} = \{0,2,-2\}$, where the causal variant is an sQTL but not an eQTL, and 4) $\mathbf{b} = \{1, 2, 3\}$, where the causal variant is both an sQTL and an eQTL. We generated the transcript abundance as $y_{ij} = x_j b_i + e_{ij}$ where y_{ij} is the transcript abundance of isoform i in individual j, x_j is the genotype of the causal variant of individual j, b_i is the effect size of the causal variant on isoform i, and e_{ij} is the residual with its variance denoted by $\mathrm{var}(e_j)$. Considering that in reality RNA-seq read counts usually follow a Poisson distribution and that expression levels of different isoforms of a gene are often correlated, we generated residuals of the three isoforms of each individual (denoted by $\mathbf{e}_j = \{e_{1j}, e_{2j}, e_{3j}\}$) from a multivariate Poisson distribution with mean $\mathbf{0}$ and variance-covariance matrix $\mathbf{S}$. The kl-

th element of $\mathbf{S}$ is $S_{kl} = r_e\sqrt{\text{var}(e_{kj})\text{var}(e_{lj})}$, where r_e is the residual correlation (sampled at random from the observed correlations of transcription abundance between isoforms in the ROSMAP data) and $\text{var}(e_{kj}) = 2p(1-p)b_k^2(\frac{1}{q_k^2} - 1)$ with p being the MAF of the causal variant and q_k^2 being the proportion of variance in transcript abundance of isoform k explained by the causal variant (which was set 10% for all the three isoforms). We used this formula to generate the residual variance to ensure the proportion of variance in the transcription abundance explained by the causal variant to be q^2. We added a sufficiently large positive mean value to the simulated transcription abundance of each isoform across all the individuals to ensure all the isoform abundances to be positive. The simulated RNA-seq data were normalized using the rank-based inverse normal transformation (RINT) and then fitted in a linear regression model to estimate isoform-eQTL effects. We also generated residuals from a multivariate normal distribution for comparison, i.e., $\mathbf{e}_j \sim MVN(\mathbf{0}, \mathbf{S})$. We repeated each simulation scenario with 500 replicates.

Apart from simulating transcriptional abundances numerically based on a hypothetical distribution, we also performed simulations in which transcriptional abundances were generated by sampling RNA-seq reads from reference sequence using Polyester[8] to mimic real RNA-seq data. This simulation setting retains the correlations of transcriptional abundance among isoforms and facilitates the comparison between methods that require different types of RNA-seq input data (e.g., TPM or read count). To simulate a scenario that takes overdispersion into account, we applied the function *simulate_experiment*() in Polyester to generate RNA-seq reads. The built-in transcript read count model assumes that the number of reads for each transcript is drawn from a negative binomial distribution across biological replicates. The parameter "*size*" in *simulate_experiment*() can be used to control the negative binomial variance, i.e., *mean* + *mean*^{2}/*size*, where *size* = *readspertx* * *fold _changes* / *s*, with *readspertx* being the read count per transcript and *fold _changes* being the fold change of transcription abundance between groups. Thus, large *s* leads to small *size* and large negative binomial variance. In this case, the RNA-seq data (as measured by TPM) are distributed with overdispersion and deviated strongly from multivariate normality. To simulate a null model where the causal variant is not an sQTL, we set the transcriptional abundances of the individuals in the three genotype groups to $1X$, $2X$, and $3X$, respectively for all the isoforms. To simulate a causal model where the causal variant is an sQTL, we set the transcriptional abundances of the individuals in the three genotype groups to 10X, 11X, $12X$ for one isoform and $12X$, $11X$, and $10X$ for the other isoforms. Note that X can, in principle, take any ordinary value; we set $X = 1$ in our simulation. We tested the performance of THISTLE in a range of simulation scenarios with varying parameters including sample size (300, 600, 900,

or 1200), sQTL effect size (small, median, or large), number of isoforms per gene (2, 5, 10, 15, or 20), and degree of over-dispersion of transcription abundance (s = 600, 900, 1200, or 1500). The sQTL effect size was measured by the maximum difference in relative isoform expression between genotype groups (MD)[9]. We simulated small (MD < 0.05), median (0.05 < MD < 0.15), or large (MD > 0.15) effects based on the sQTLseekeR results from the ROSMAP dataset. Once RNA-seq reads were simulated, we mapped reads from each sample using STAR[10] to GRCh37, applied RSEM[11] to quantify isoform expression levels, and ran an sQTL analysis using THISTLE. We have also included sQTLseekeR, DRIMSeq[12], and MANOVA into the comparison. Each simulation scenario was repeated 500 times.

The false-positive rate (FPR) was computed as the proportion of significant variants (e.g., $P < 0.05$) among the simulated null variants across 500 simulation replicates. True positive rate (TPR) was calculated as the proportion of simulation replicates in which the sQTL effect was detected at a given significance level (e.g., $P < 0.05$). Considering potential inflation in FPR of a method under the null, we also compared the sQTL methods by AUC at the same level of FPR (i.e., 0.05 or 0.001).

3. Comparing THISTLE with sQTLseekeR in real data analysis

We compared THISTLE with sQTLseekeR[3,9] in the analysis of the ROSMAP data. Following the analysis pipeline provided by the sQTLseekeR authors[3,9], we included in the analysis genes with splicing dispersion > 0.01 and more than 25 splicing patterns. For each gene, only the individuals with TPM > 0.1 were included in the sQTL test. To be consistent with the THISTLE analysis, we included in the sQTLseekeR analysis only the SNPs located in the gene in query or within 2 Mb upstream or downstream of the gene. In total, 14,412 genes and 7,384,172 SNPs that were tested in both sQTLseekeR and THISTLE analyses were included in the comparison.

4. Principal component analysis

To identify individuals of European ancestry from our samples, we performed a principal component analysis (PCA) of each cohort with the sequence-based genotype data from the 1000 Genomes Project[13] (1KGP; n = 2,504), comprising individuals of European (EUR), East Asian (EAS), Admixed American (AMR), South Asian (SAS), and African (AFR) ancestries. Only the autosomal SNPs with missingness rate <5%, MAF >1% and available in HapMap3[14] and individuals with missingness rate <5% were included in the PCA. After the PCA, we removed the individuals whose principal component 1 (PC_1) or PC_3 deviated more than 6 standard deviations from the mean of the corresponding PC of the 1KGP-EUR.

5. A permutation-based procedure for multiple testing correction for cis-sQTLs or cis-eQTLs

For the cis-eQTLs, we shuffled the gene-level transcriptional abundance phenotype across individuals and re-ran the eQTL analysis with QTLtools. For the LeafCutter & QTLtools cis-sQTLs, we shuffled the splicing phenotype (using the --grp-best option in QTLtools) across individuals and re-ran the sQTL analysis with QTLtools. For the THISTLE sQTLs, we permuted the isoform-level transcriptional abundance phenotype across individuals and re-ran the isoform-eQTL mapping and the THISTLE sQTL analysis using OSCA. In each of the three scenarios above, we ran 1,000 permutations and fitted a beta distribution to the minimum p-values obtained from the permutations to compute the effective number of independent tests (m_e) for each gene. We adjusted the nominal sQTL or eQTL p-values by m_e and employed the Storey's q-value approach[15] to the lead sQTL or eQTL SNPs to account for multiple testing across genes. We performed this permutation procedure in each of the ten datasets.

6. Comparison to previous sQTL studies

There were a few studies[16-19] which also mapped the sQTLs in brain. The differences in the numbers of sGenes and unique sQTL SNPs (without clumping) detected between our study and the previous studies[16-19] are remarkable, reflecting the differences in the sample size (**Supplementary Table 4)**. Among all the previous sQTL studies, the Wang et al. study[19] has the largest sample size, which, however, is still more than two times smaller than our sample size. Apart from the difference in sample size, there is a difference in methodology (**Supplementary Table 4)**. We have shown that a combination of THISTLE and LeafCutter maximizes the sQTL detection power (**Extended Data Figure 7**). However, all the previous studies, including the Wang et al. study, only used a single sQTL method. There were two versions of the Wang et al. sQTL summary statistics available, with one version excluding transcripts whose expression levels were < 5 FPKM in all the individuals and the other version without such filtering. Under the same criterion without filtering transcripts, we identified 13,716 sGenes (with $P_{sQTL} < 5 \times 10^{-8}$) with 1,875,864 unique sQTL SNPs (without clumping), compared to 7,296 sGenes and 462,722 unique sQTL SNPs in the Wang et al. study, 1.7- and 4-fold increase in the numbers of sGenes and sQTL SNPs, respectively (**Supplementary Figure 9** and **Supplementary Table 4**). Linking the sQTLs to the GWAS signals for the 12 complex traits, the number of genes passing the SMR & COLOC PP4 thresholds was 255 using our data and 146 using the Wang et al. data, a 1.7-fold increase.

While previous studies have focused on identifying genes for which the sQTL signals are colocalized with the GWAS signals for a few specific diseases, our study was aimed at quantifying

the role of sQTLs, in comparison to eQTLs, in mediating genetic effects for a host of complex traits using powerful datasets.

7. Sampling variance of the estimated fold enrichment

Let x represent the estimated per-SNP heritability for the SNPs in query, and $\boldsymbol{y} = \{y_1, y_2, \dots, y_j, \dots, y_m\}$ with y_j being the corresponding estimate for the control SNPs in the j_{th} replicate (noted that the control SNPs are randomly sampled with MAF and genomic location matched with the SNPs in query). The fold enrichment is calculated as $x/\bar{y}$, where $\bar{y}$ is the mean across all the elements of $\boldsymbol{y}$. The variance of $x/\bar{y}$ can be computed approximately by the Delta method[20],

$$\mathrm{var}\left(\frac{x}{\bar{y}}\right) \approx \left(\frac{x}{\bar{y}}\right)^2 [\frac{\mathrm{var}(x)}{x^2} + \frac{\mathrm{var}(\bar{y})}{\bar{y}^2} - \frac{2\mathrm{cov}(\mathrm{x}, \bar{y})}{x\bar{y}}]$$

If we assume the covariance between x and $\bar{y}$ is 0 and the variance of x is $\mathrm{var}(x) \approx \widehat{\mathrm{var}}(y)$, where $\widehat{\mathrm{var}}(y)$ is the observed variance of y across m replicates, the variance of fold enrichment can be approximated by

$$\mathrm{var}\left(\frac{x}{\bar{y}}\right) \approx \left(\frac{x}{\bar{y}}\right)^2 [\frac{\mathrm{var}(x)}{x^2} + \frac{\mathrm{var}(\bar{y})}{\bar{y}^2}] \approx \left(\frac{x}{\bar{y}}\right)^2 [\frac{\widehat{\mathrm{var}}(y)}{x^2} + \frac{\widehat{\mathrm{var}}(y)}{m\bar{y}^2}]$$

8. The advantages of using two complementary strategies for sQTL mapping

Here are several reasons why the two strategies may complement each other. First, there is uncertainty for transcript-level analysis in quantifying transcriptional abundance from short-read RNA-seq data given ubiquitous overlaps among transcript isoforms, which, however, is not an issue for event-level method, such as LeafCutter, as it detects intron excision events locally from mapped split reads. Second, event-level analysis focuses on local intron excision events and thus may not capture the complete landscape of alternative splicing, whereas transcript-level analysis is benefited from using the annotated transcripts from the reference transcriptome and thus can capture complex splicing events, retained introns, and alternative UTRs (**Supplementary Figure 3**). Third, LeafCutter is reference-free, thereby not limited to known isoforms. Fourth, although THISTLE and LeafCutter & QTLtools identified a similar number of sGenes (9,305 vs. 8,602; **Extended Data Figure 7a**), the number of genome-wide significant trait-associated genes discovered by SMR & COLOC PP4 through the THISTLE sQTLs was much larger than that through the LeafCutter & QTLtools sQTLs (184 vs. 114; Supplementary Table 6), partly because the Bonferroni correction in the SMR analysis using the LeafCutter & QTLtools sQTL summary data (m = ~44,000 tests for each trait) was more conservative than that using the THISTLE sQTL summary data (m = ~8,600).

9. Limitations of this study

Our study has several limitations. First, the transcript-level sQTL analysis relies on accurate assembly and quantification of mRNA isoforms, which are limited by the short-read RNA-seq technology but could be improved in the future by long-read RNA-seq. For example, the Pacific Biosciences (PacBio) isoform sequencing technology has identified many novel transcripts and alternative splicing events in a range of tissues and cell types[21]. Second, the sQTLs and eQTLs identified in this study only explain a small fraction of the GWAS loci for the twelve traits, and most of the loci remain unexplained, likely due to the insufficient sample size of the transcriptome data and mechanisms beyond genetic control of transcriptional regulation. Thus, a comprehensive set of genetic variants associated with other molecular phenotypes (e.g., chromatin accessibility, DNA methylation, histone modification, and protein abundance) from studies with very large sample sizes is essential to unveil the molecular basis of polygenic trait variation. Third, we have identified many genetic variants associated with mRNA abundance and pre-mRNA splicing, but the causative functional variants are mostly unknown. Fine-mapping analyses and functional experiments are required to pinpoint the causal variants and elucidate the mechanisms behind genetic control of transcriptional regulation and the following cascades of events that lead to phenotypic changes. Fourth, THISTLE is a heterogeneity test, which does not produce the effect size of an sQTL, limiting follow up analyses that requires such information. To mitigate this limitation, we report the isoform-eQTL effects for the significant sQTLs from THISTLE. Finally, this study focuses on brain transcriptomic data and brain-related traits. Although it is likely that our conclusions can be generalized to other tissues and complex traits as indicated by our analysis with seven additional traits (**Supplementary Figure 22**), the generalization of our conclusions needs to be confirmed by well-powered studies in the future.

10. Acknowledgments

PsychENCODE

Data were generated as part of the PsychENCODE Consortium supported by: U01MH103339, U01MH103365, U01MH103392, U01MH103340, U01MH103346, R01MH105472, R01MH094714, R01MH105898, R21MH102791, R21MH105881, R21MH103877, and P50MH106934 awarded to: Schahram Akbarian (Icahn School of Medicine at Mount Sinai), Gregory Crawford (Duke), Stella Dracheva (Icahn School of Medicine at Mount Sinai), Peggy Farnham (USC), Mark Gerstein (Yale), Daniel Geschwind (UCLA), Thomas M. Hyde (LIBD), Andrew Jaffe (LIBD), James A. Knowles (USC), Chunyu Liu (UIC), Dalila Pinto (Icahn School of Medicine at Mount Sinai), Nenad Sestan (Yale), Pamela Sklar (Icahn School of Medicine at Mount Sinai), Matthew State (UCSF), Patrick Sullivan (UNC), Flora Vaccarino (Yale), Sherman Weissman (Yale), Kevin White (UChicago) and Peter Zandi (JHU).

ROSMAP: Study data were provided by the Rush Alzheimer's Disease Center, Rush University Medical Center, Chicago. Data collection was supported through funding by NIA grants P30AG10161 (ROS), R01AG15819 (ROSMAP; genomics and RNAseq), R01AG17917 (MAP), R01AG30146, R01AG36836 (RNAseq), R01AG48015 (monocyte RNAseq), RF1AG57473 (single nucleus RNAseq), U01AG32984 (genomic and whole exome sequencing), U01AG61356 (whole genome sequencing, targeted proteomics, ROSMAP AMP-AD), the Illinois Department of Public Health (ROSMAP), and the Translational Genomics Research Institute (genomic).

MSBB: These data were generated from postmortem brain tissue collected through the Mount Sinai VA Medical Center Brain Bank and were provided by Dr. Eric Schadt from Mount Sinai School of Medicine.

Mayo: The Mayo RNAseq study data was led by Dr. Nilüfer Ertekin-Taner, Mayo Clinic, Jacksonville, FL as part of the multi-PI U01 AG046139 (MPIs Golde, Ertekin-Taner, Younkin, Price). Samples were provided from the following sources: The Mayo Clinic Brain Bank. Data collection was supported through funding by NIA grants P50 AG016574, R01 AG032990, U01 AG046139, R01 AG018023, U01 AG006576, U01 AG006786, R01 AG025711, R01 AG017216, R01 AG003949, NINDS grant R01 NS080820, CurePSP Foundation, and support from Mayo Foundation. Study data includes samples collected through the Sun Health Research Institute Brain and Body Donation Program of Sun City, Arizona. The Brain and Body Donation Program is supported by the National Institute of Neurological Disorders and Stroke (U24 NS072026 National Brain and Tissue Resource for Parkinsons Disease and Related Disorders), the National Institute on Aging (P30 AG19610 Arizona Alzheimers Disease Core Center), the Arizona Department of Health Services (contract 211002, Arizona Alzheimers Research Center), the Arizona Biomedical Research Commission (contracts 4001, 0011, 05-901 and 1001 to the Arizona Parkinson's Disease Consortium) and the Michael J. Fox Foundation for Parkinsons Research.

Supplementary Figures

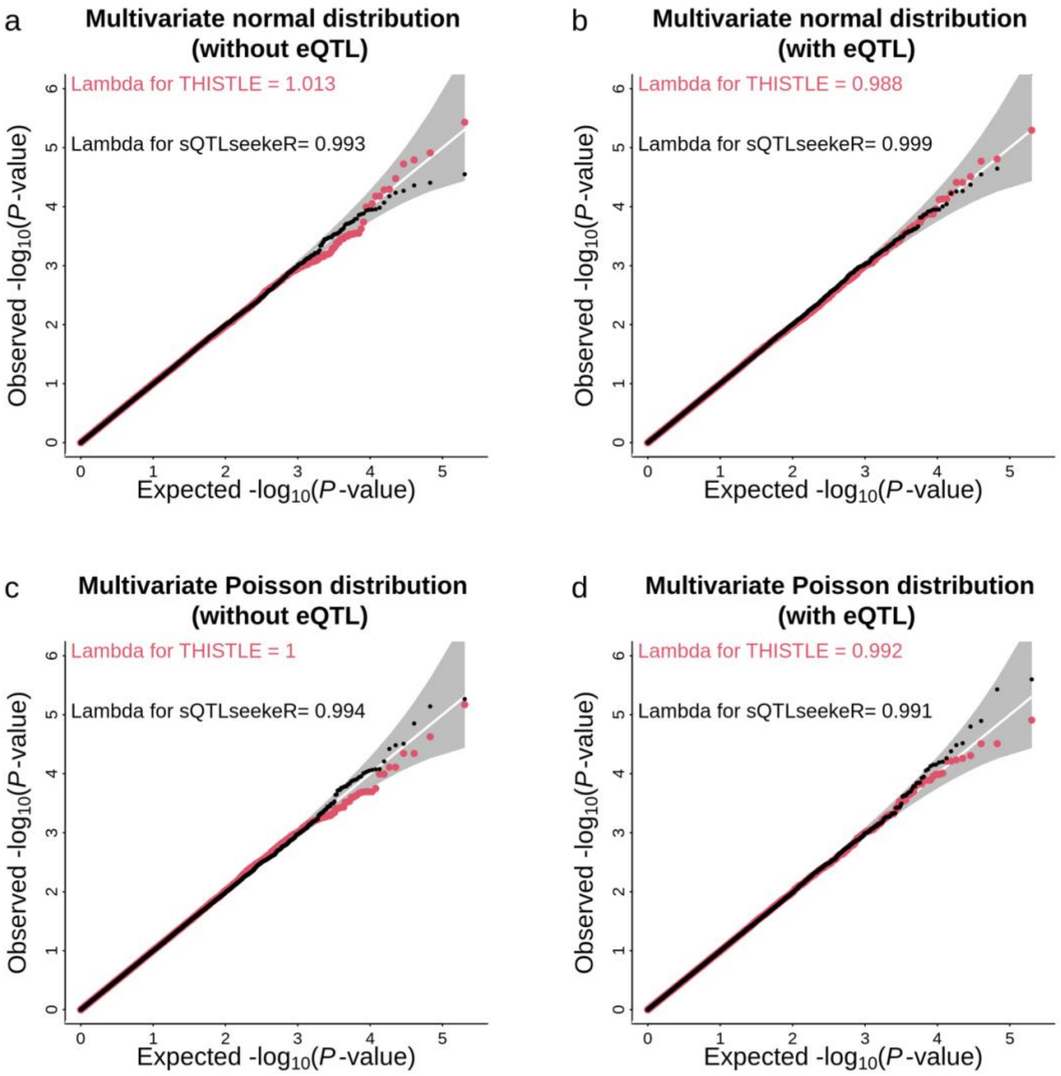

Supplementary Figure 1. Quantile-quantile (QQ) plots for sQTL analysis under the null of no sQTL effect using THISTLE (red) and sQTLseekeR (black). Panels **a** and **b** show the QQ plots for sQTL analysis with the transcription abundance simulated from a multivariate normal distribution (with or without eQTL effect). Panels **c** and **d** show the QQ plots for sQTL analysis with the transcription abundance simulated from a multivariate Poisson distribution. The grey shaded area represents the 95% confidence interval (CI). The THISTLE and sQTLseekeR p-values were computed using a one-sided sum of chi-squared test (approximated by the Saddlepoint algorithm) and pseudo-*F* test (approximated by the Davies algorithm), respectively.

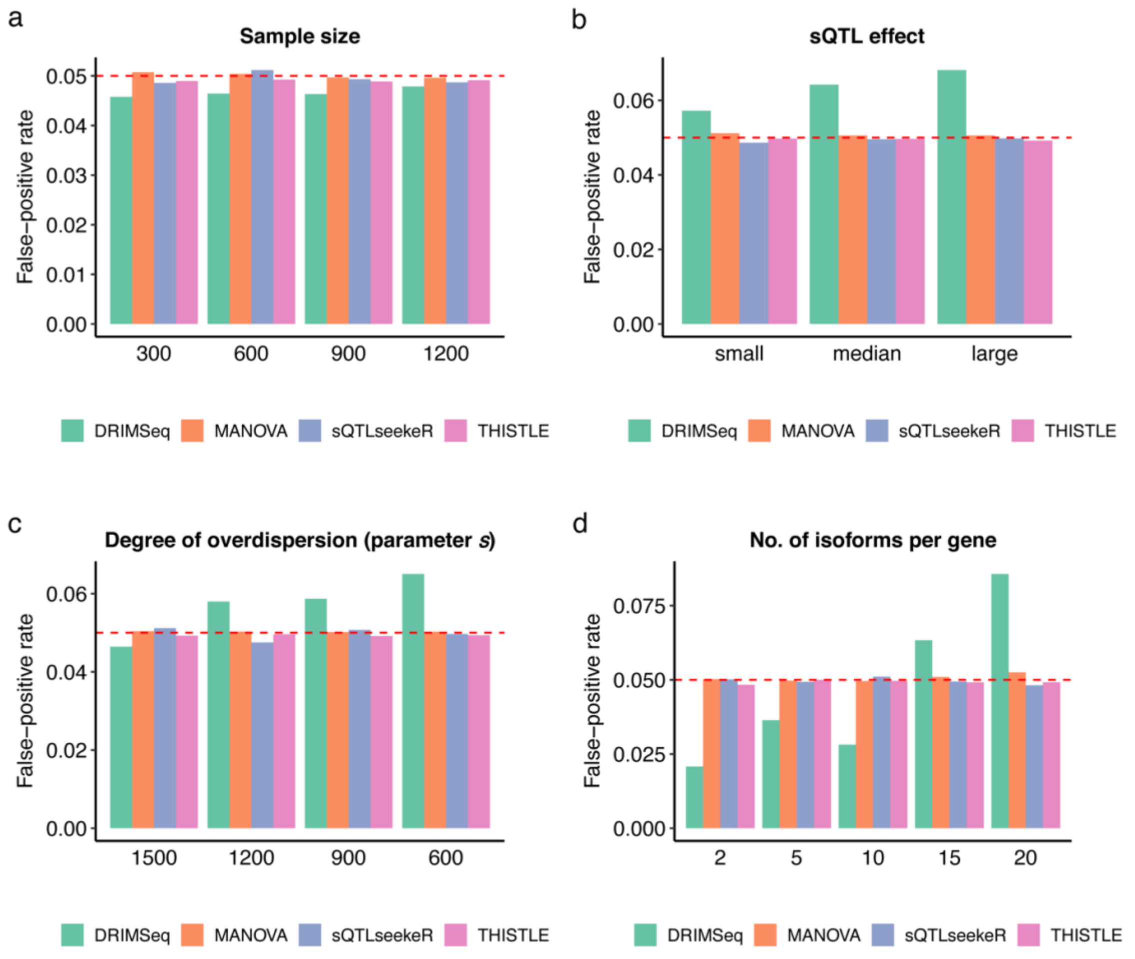

Supplementary Figure 2. FPR for DRIMSeq, MANOVA, sQTLseekeR, and THISTLE in simulations. Shown are the results from 500 simulation replicates to quantify the FPR for DRIMSeq, MANOVA, sQTLseekeR, and THISTLE. The mRNA abundances were generated by sampling RNA-seq reads to mimic real RNA-seq data using Polyester (section 2 of the **Supplementary Note**). The simulations were performed with varying **a**) sample size, **b**) sQTL effect size, **c**) the degree of overdispersion of transcription abundances, and **d**) the number of isoforms per gene. When one simulation parameter was fixed (e.g., n = 300 in panel **a**), all the other parameters were randomly sampled from the specified categories (e.g., the sQTL effect size from {small, median, or large}, the number of isoforms per gene from {2, 5, 10, 15, or 20}, and the degree of over-dispersion of transcriptional abundance from {600, 900, 1200, or 1500}). FPR was computed as the proportion of SNPs with p-value less than a threshold (e.g., $P_{\mathrm{sQTL}} < 0.05$) among the null variants across 500 simulation replicates. The red dashed line represents an FPR of 0.05. The DRIMSeq, and MANOVA, sQTLseekeR, and THISTLE p-values were computed using a one-sided likelihood ratio test, F test, pseudo-F test, and sum of chi-squared test, respectively.

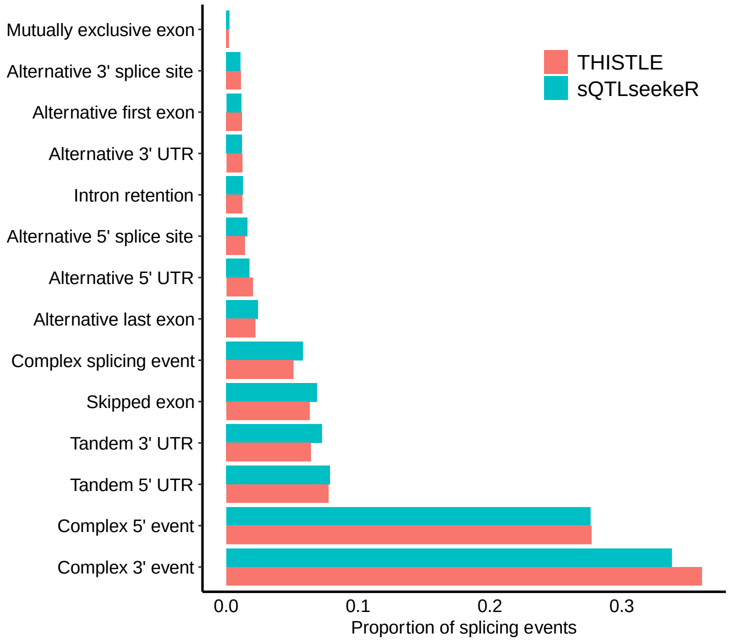

Supplementary Figure 3. Distribution of the splicing events for the sGenes identified by THISTLE and sQTLseekeR from the ROSMAP data. We identified the splicing event(s) for each sGene by comparing the isoforms showing the most positive and negative isoform-eQTL effects at the lead sQTL SNP, using the pipeline available at the sQTLseekeR website (https://github.com/guigolab/sQTLseekeR/blob/master/docs/SplicingEventClassification.md). Note that sGenes for which the isoform-eQTL effects of the lead sQTL SNP were in a consistent direction were discarded.

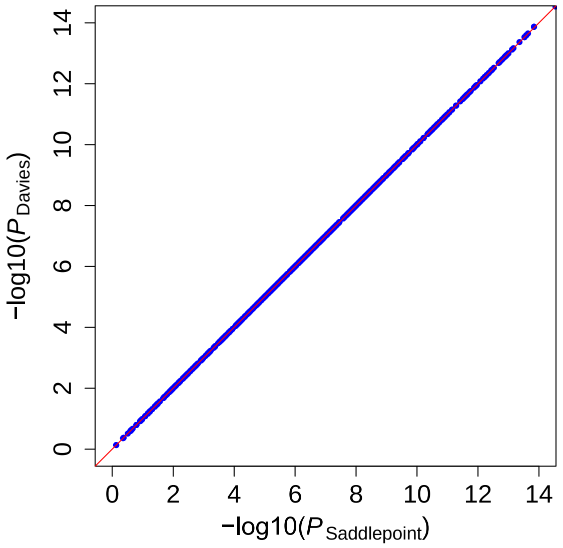

Supplementary Figure 4. Comparison of THISTLE p-values computed using the Saddlepoint approximation with those using the Davies method in simulations. We simulated genotype data for unlinked SNPs from binomial distributions and sampled a SNP at random as an sQTL (see scenario #3 in section 2 of the **Supplementary Note** for details). We ran THISTLE with sQTL p-values computed from a one-sided sum of chi-squared test using either the Saddlepoint or Davies approximation. We repeated the simulation 1,000 times. Shown are the p-values (pooled across simulation replicates) truncated at 1×10^{-14}, a precision limit for the Davies algorithm suggested in Diego Garrido-Martín *et al.*[9]. The red dashed line is the diagonal line.

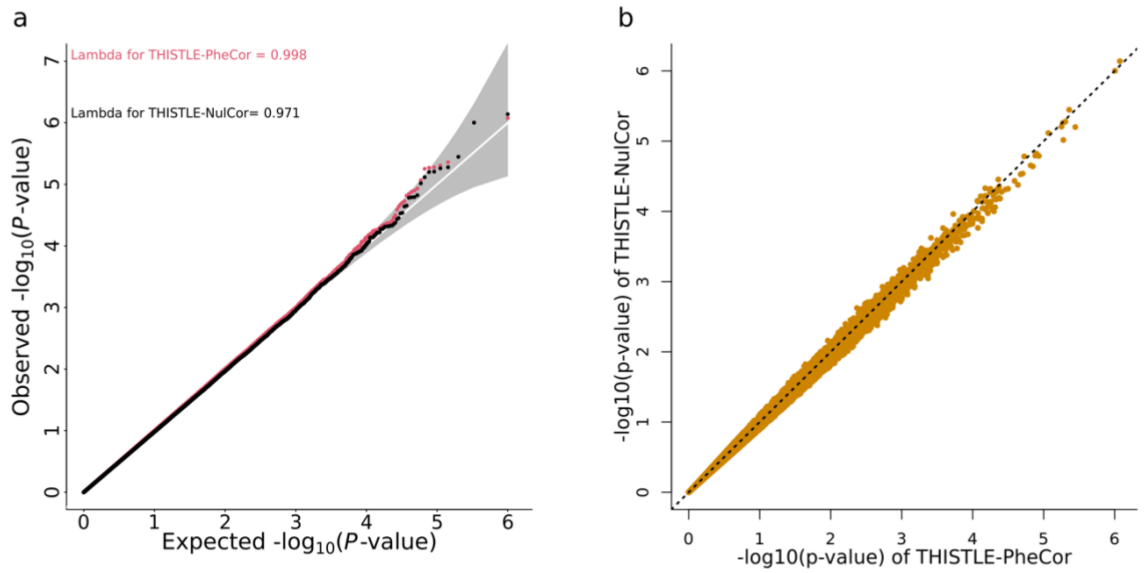

Supplementary Figure 5. Comparison between individual-level data- and summary-level data-based THISTLE analyses. The only difference between the individual-level data- and the summary-level data-based THISTLE analyses is how the sampling correlation (θ_{jk}) in the estimated SNP effect between two isoforms is obtained. **a**) QQ plot under the null (see scenario #2 in section 2 of the **Supplementary Note** for details) for THISTLE-PheCor (θ_{jk} estimated from observed phenotypes; coloured in red) and THISTLE-NulCor (θ_{jk} estimated from SNPs with $P_{isoform\text{-}eQTL}$ > 0.01 using summary data; coloured in black). The grey shaded area represents the 95% CI. **b**) Comparison of association statistics between THISTLE-PheCor and THISTLE-NulCor. The black dashed line is the diagonal line. The THISTLE p-values were computed from the one-sided sum of chi-squared test.

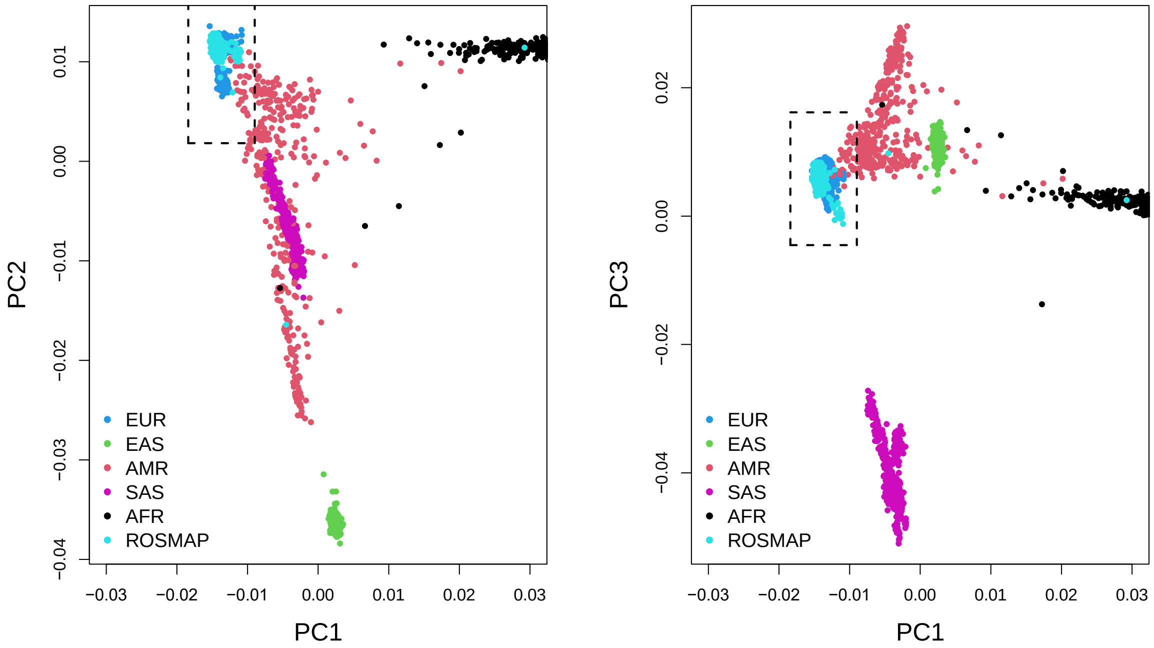

Supplementary Figure 6. Principal component analysis (PCA). We investigated the population structure of each cohort by PCA with data from the 1000 Genomes Project (1KGP) (n = 2,504), comprising individuals of European (EUR), East Asian (EAS), Admixed American (AMR), South Asian (SAS), and African (AFR) ancestries. Here is a demonstration with the ROSMAP cohort (n = 844). The dashed line rectangle represents +/- 6 standard deviations from the mean PC of 1KGP-EUR.

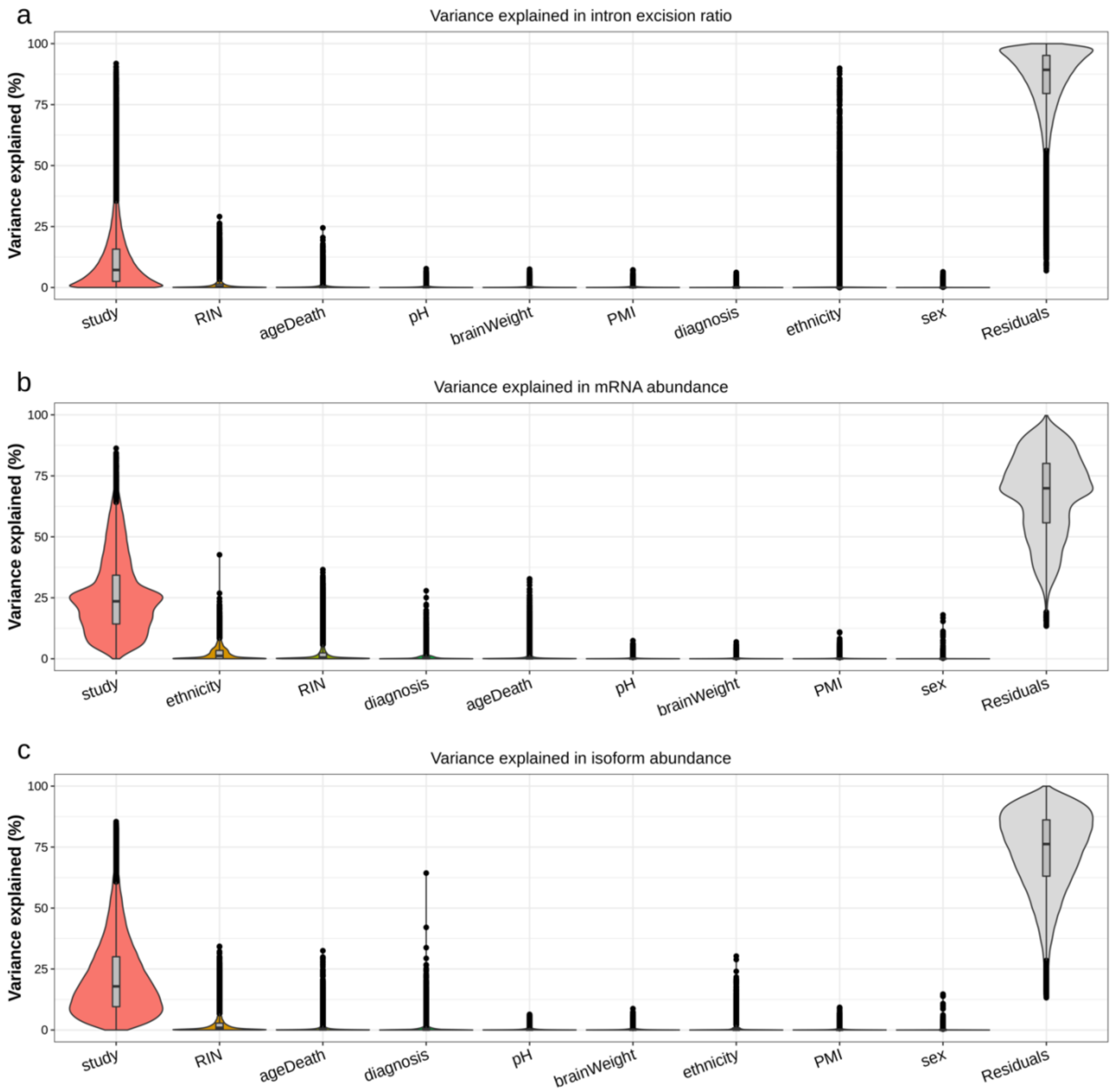

Supplementary Figure 7. Violin and box plots of the proportion of variation in intronic excision ratio (**a**), mRNA abundance (**b**), or isoform abundance (**c**) explained by the biological and technical factors using variancePartition[22]. The factors include study cohort (e.g., BrainGVEX, LIBD, CMC, or HBCC), RIN (RNA integrity number), ageDeath (age of death), pH, brain weight, ethnicity, PMI (post-mortem interval), sex, and diagnosis (i.e., SCZ, BIP, ASD, or control). We performed the variancePartition in each of the 10 datasets (**Supplementary Table 1**). Shown are the results pooled across the four PsychENCODE datasets, i.e., BrainGVEX (*n* = 384), LIBD (*n* = 128), CMC (*n* = 469), or HBCC (*n* = 129). Each violin plot shows the distribution of variance explained by a factor for 273,051 introns, 31,451 genes, or 163,353 isoforms in panel **a**, **b**, or **c**, respectively. The line inside each box indicates the median value, notches indicate the 95% CI, the central box indicates the interquartile range (IQR), whiskers indicate data up to 1.5 times the IQR, and outliers are shown as separate dots.

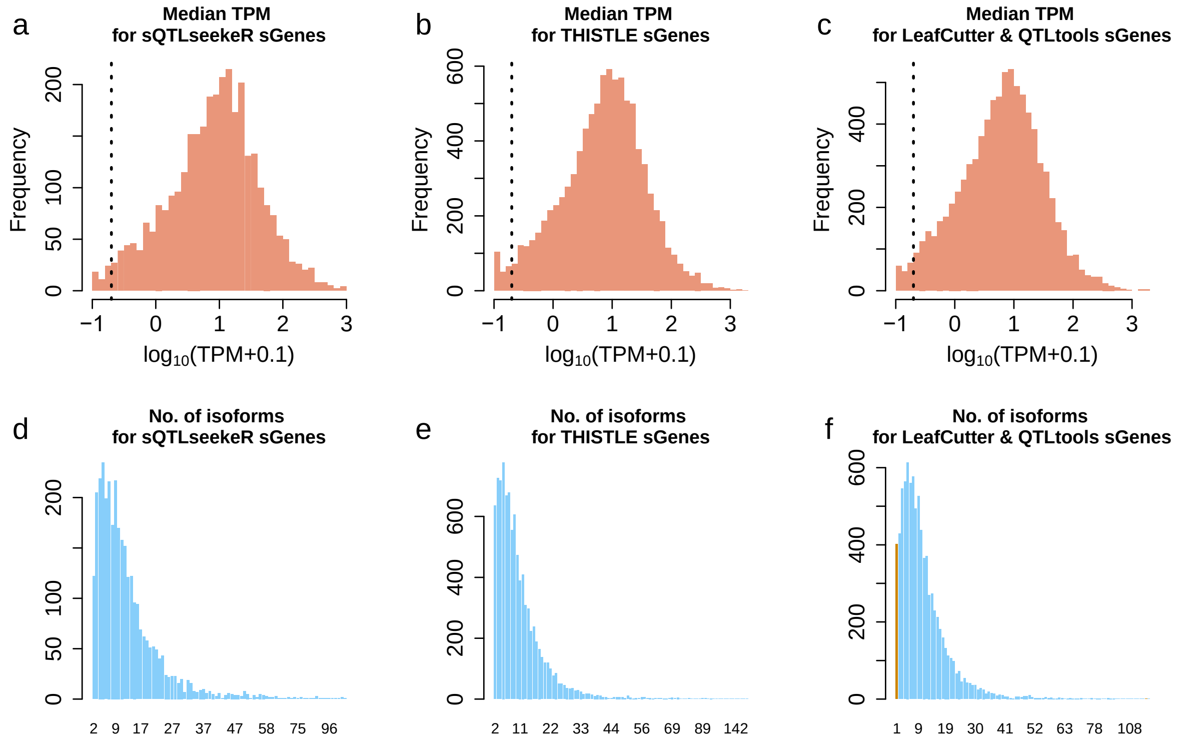

Supplementary Figure 8. Expression level and number of isoforms of the sGenes identified by sQTLseekeR (**a** and **d**), THISTLE (**b** and **e**) and LeafCutter & QTLtools (**c** and **f**) in the ROSMAP data. TPM: transcripts per million. In panels **a**, **b**, and **c**, each value is the median of gene-level transcriptional abundances across individuals, and the black dashed lines represent a TPM value of 0.1. The orange bar in panel **f** represents isoform number of 1.

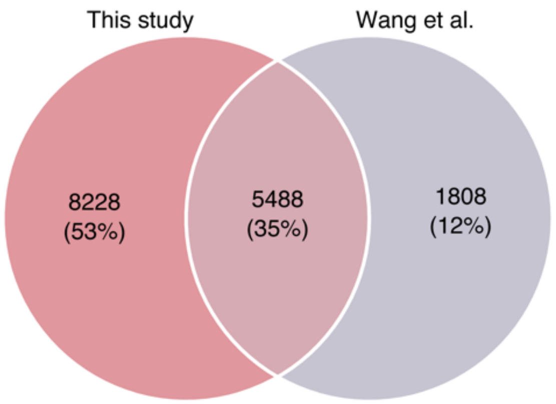

Supplementary Figure 9. Comparison between the sGenes identified in this study and the Wang et al. study[19]. The sQTLs in the two studies were identified under the same quality control criterion, i.e., without excluding lowly expressed transcripts.

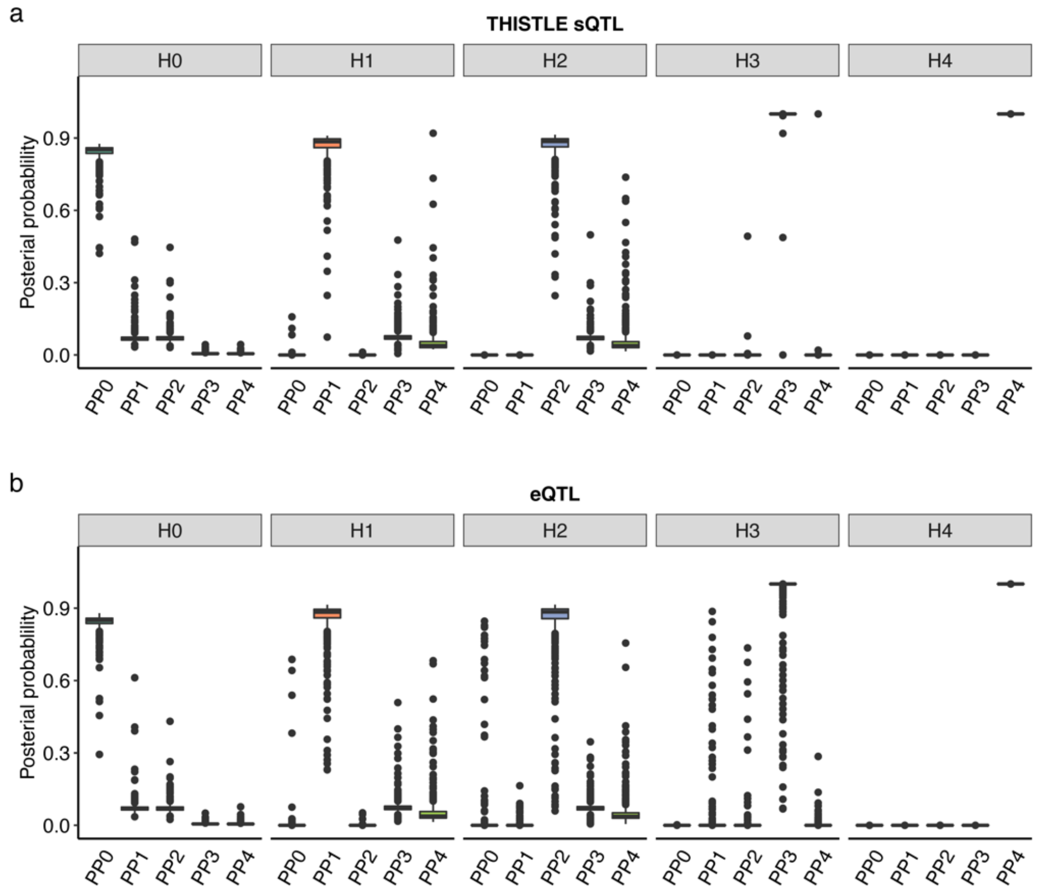

Supplementary Figure 10. Demonstrating the validity of using THITSLE sQTL p-values in COLOC analysis by simulation. We simulated genotype data for unlinked SNPs from binomial distributions using the method described in section 2 of the **Supplementary Note**. Two splicing phenotypes (SPs), with each SP consisting of the abundances of two isoforms of a hypothetical gene, were simulated under 5 scenarios fulfilling the five COLOC hypotheses (H0-H4). H0: no sQTL for either SP. H1: an sQTL affecting SP 1 but not SP 2. H2: an sQTL affecting SP 2 but not SP 1. H3: an sQTL for SP 1 and another distinct sQTL for SP 2. H4: a shared sQTL for both SPs. Each scenario was repeated 500 times. To set a benchmark for comparison, we also ran 500 simulations for eQTL analysis with the mean abundance of the two isoforms as the phenotype. The THISTLE sQTL p-values were generated from the one-side sum of chi-squared test using the Saddlepoint approximation, and the eQTL p-values were generated from linear regression (one-sided chi-squared test). Each boxplot represents the distribution of a COLOC posterior probability (PP0, PP1, PP2, PP3, or PP4) across 500 simulation replicates. The line inside each box indicates the median value, notches indicate the 95% CI, the central box indicates the IQR, whiskers indicate data up to 1.5 times the IQR, and outliers are shown as separate dots. The result demonstrates that there is no apparent difference in performance between COLOC with THISTLE sQTLs (top five panels) and eQTLs (bottom five panels).

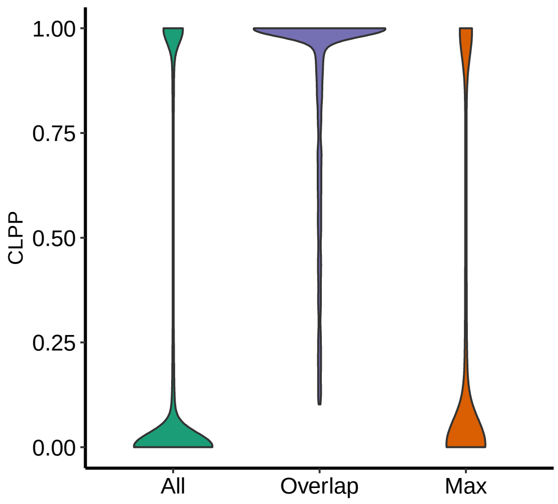

Supplementary Figure 11. Colocalization posterior probabilities (CLPP) from eCAVIAR for the sGenes that were also eGenes. CLPP is the posterior probability of a variant being an sQTL causal variant multiplied by its posterior probability being an eQTL causal variant. All: shown are the CLPP for all the variants identified as either an sQTL or an eQTL causal variant. Overlap: only the variants identified as both an sQTL and an eQTL causal variant (i.e., posterior probability > 0.1) are shown. Max: only the maximum CLPP for a gene is shown. The numbers of genes are 17,936, 4,072, and 8,968 in the three violin plots, respectively. Note that only the LeafCutter & QTLtools sQTLs were analyzed with eCAVIAR because it requires signed z-statistics which are not available for the THISTLE sQTLs.

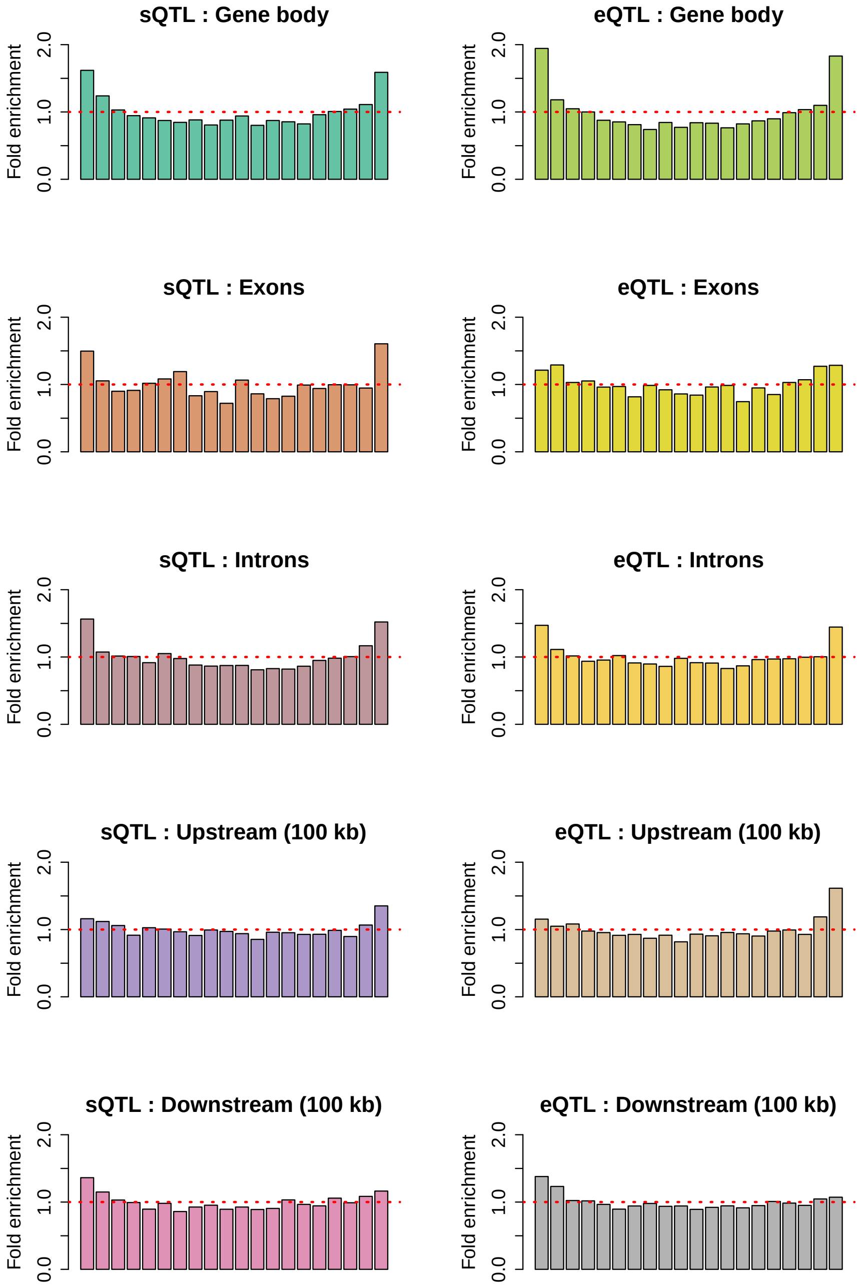

Supplementary Figure 12. Enrichment of the lead cis-sQTL and cis-eQTL SNPs in introns, exons, gene bodies, upstream regions, or downstream regions of the corresponding genes. We divided each region (e.g., exon) into 20 bins of equal size, assigned each lead xQTL SNP to the corresponding bin and computed the proportion of the lead xQTL SNPs in each bin across genes. To control for SNP ascertainment bias, we assigned all the SNPs included in the cis-xQTL analysis to each bin and computed the proportion of analyzed SNPs in each bin. The fold enrichment in each bin was computed as a ratio of the proportion of the lead xQTL SNPs to that of the analyzed SNPs.

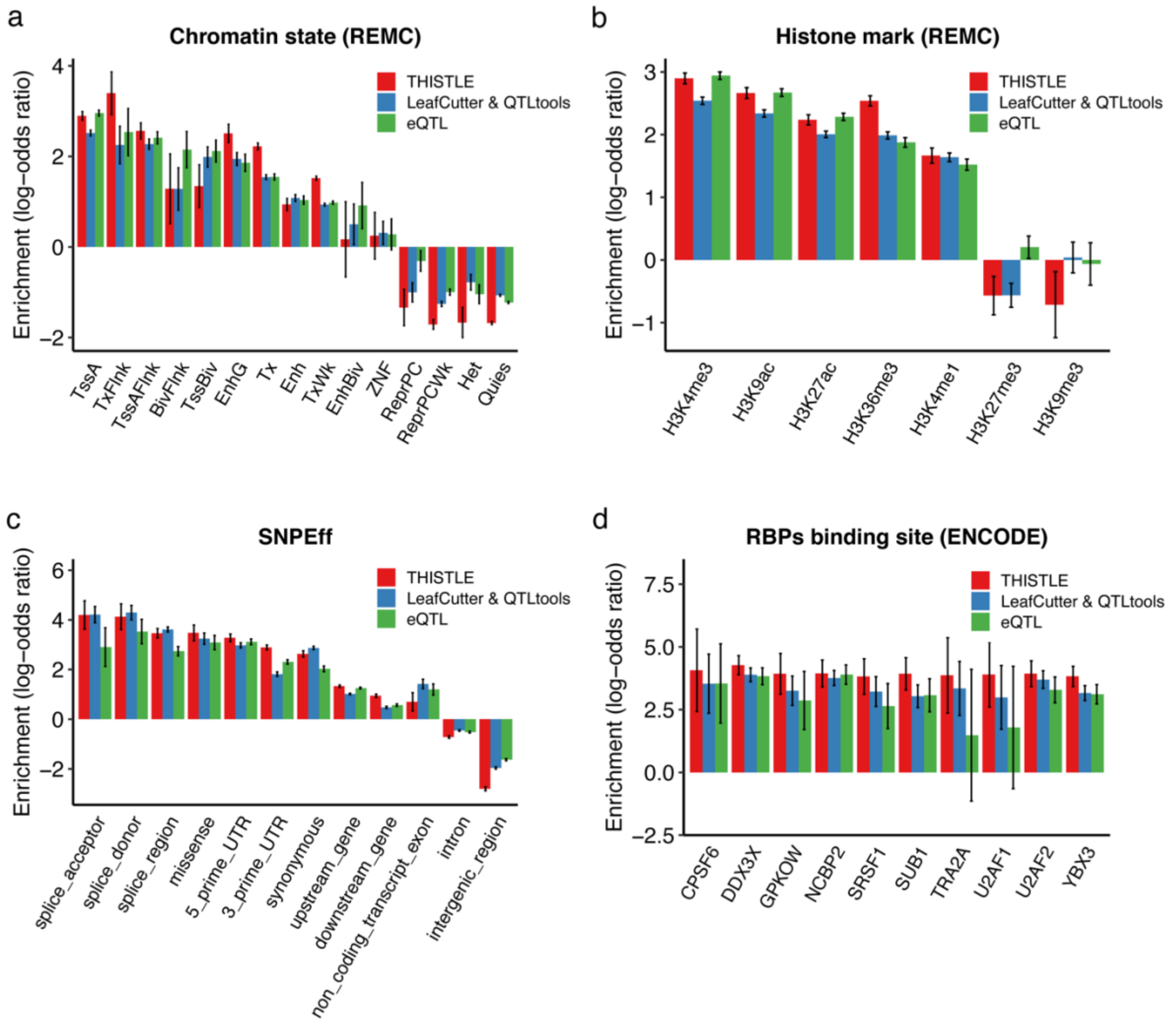

Supplementary Figure 13. Functional enrichment analysis of the cis-sQTL/eQTL SNPs without selection. The annotation categories were defined by the chromatin state annotation data from REMC (**a**), histone marks from REMC (**b**), predicted variant functions by SNPEff (**c**), or 10 selected eCLIP peaks of RBPs binding sites from ENCODE (**d**). The functional enrichment analysis was performed with TORUS using full cis-sQTL (or cis-eQTL) summary statistics without SNP selection to assess whether being in a functional category is less or more likely to be an sQTL (or eQTL) SNP. The enrichment score reported by TORUS is a logistic regression coefficient (i.e., the logarithm of odds ratio), with a positive value indicating enrichment and a negative value indicating depletion. Each column represents a point estimate of the fold enrichment with an error bar indicating the 95% CI of the estimate.

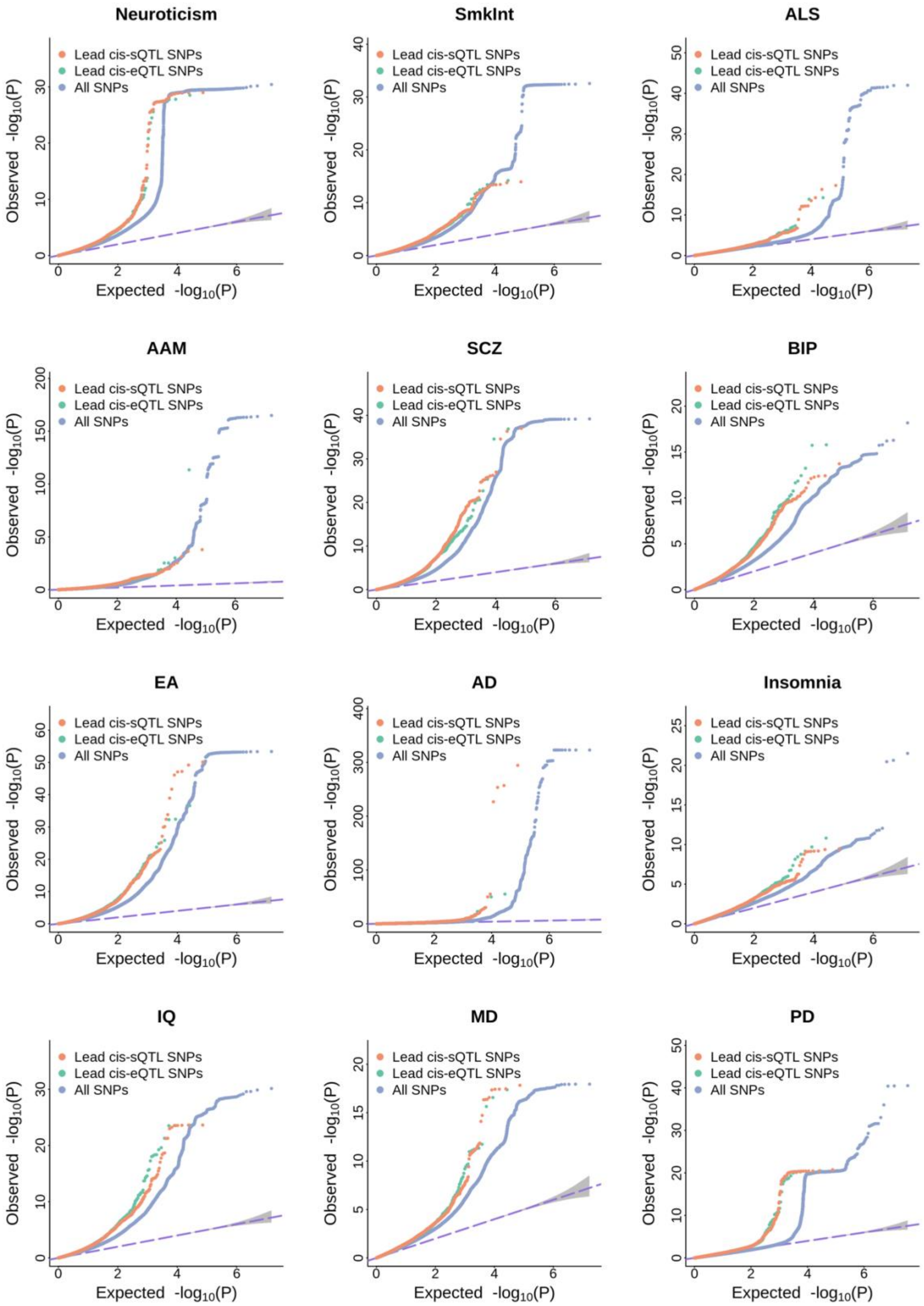

Supplementary Figure 14. QQ-plots of GWAS p-values for the twelve brain-related traits. The GWAS p-values were computed using a one-sided chi-squared test. The lead cis-sQTL and cis-eQTL SNPs are highlighted in orange and green, respectively.

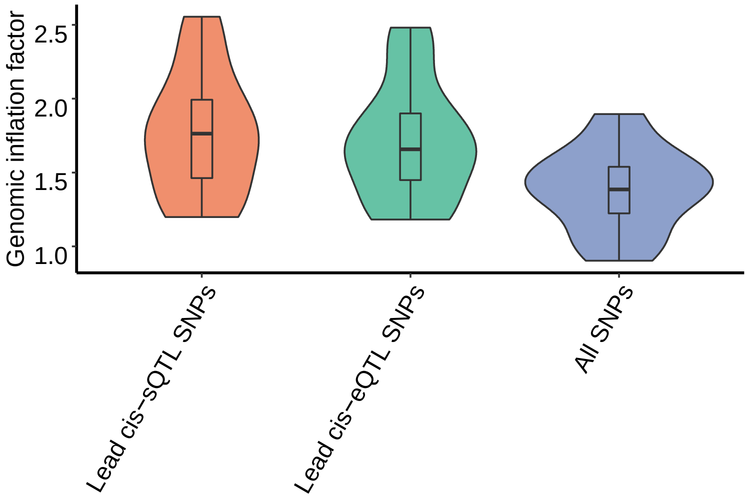

Supplementary Figure 15. Genomic inflation factors of GWAS test-statistics for the lead cis-sQTL SNPs, the lead cis-eQTL SNPs, and all SNPs, respectively, for the 12 brain-related traits. Each violin plot represents the distribution of genomic inflation factors for a set of SNPs across the 12 traits. The line inside each box indicates the median value, notches indicate the 95% CI, the central box indicates the IQR, and whiskers indicate data up to 1.5 times the IQR.

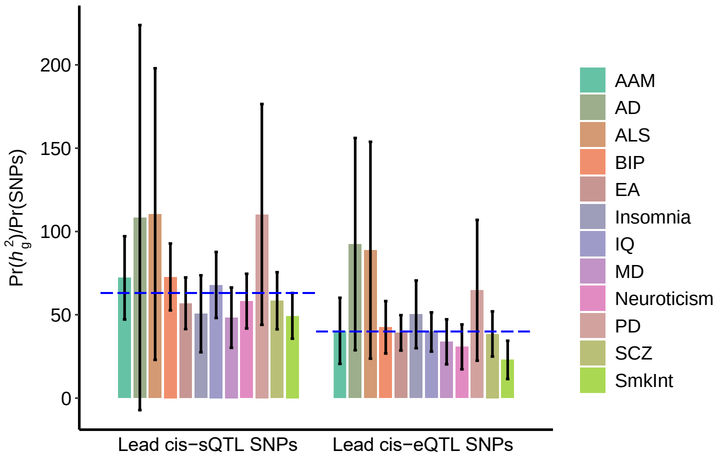

Supplementary Figure 16. Enrichment of the lead cis-sQTL or cis-eQTL SNPs for heritability of the twelve brain-related traits. Heritability enrichment is defined as a ratio of the proportion of SNP-based heritability explained by the SNPs in query to the proportion of the SNPs, i.e., $\Pr\left(h_{\mathrm{g}}^2\right)/\Pr\,(\mathrm{SNPs})$ with $\Pr\left(h_{\mathrm{g}}^2\right) = \frac{h_{\mathrm{xQTL}}^2}{h_{\mathrm{SNP}}^2}$ and $\Pr(\mathrm{SNPs}) = \frac{\#\text{ of SNPs in query}}{\#\text{ of SNPs in total}}$. The blue dashed line represents the median value across traits. Each column shows a point estimate of the fold enrichment with an error bar representing the 95% CI of the estimate.

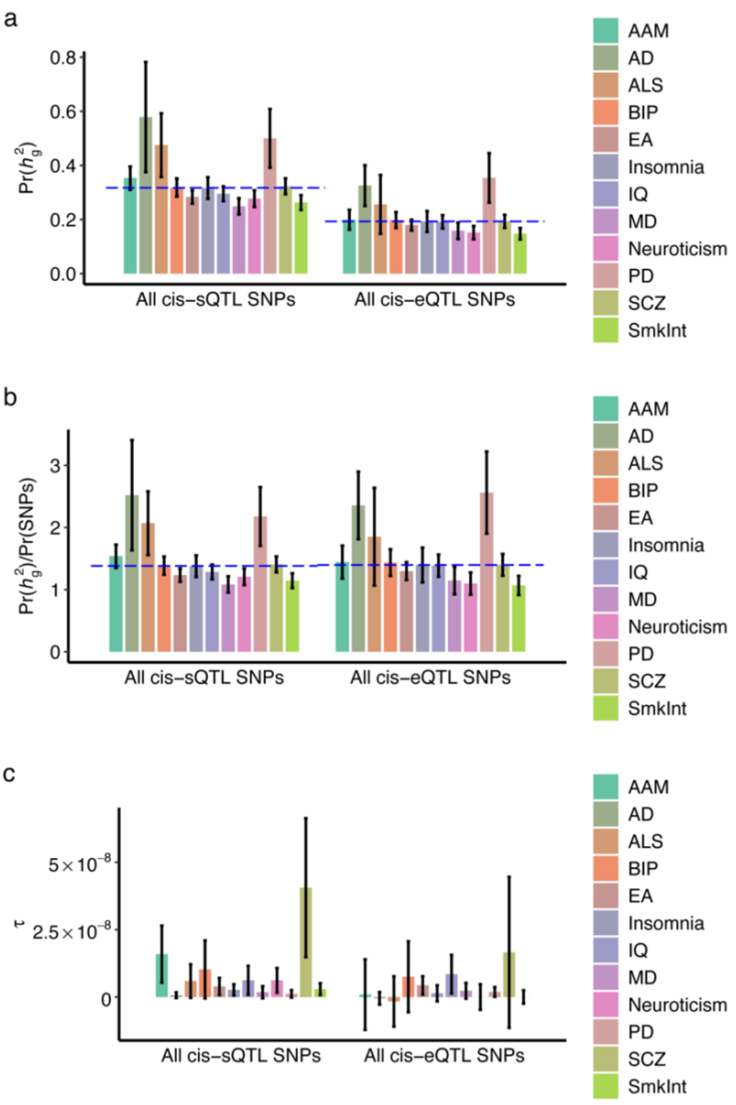

Supplementary Figure 17. Enrichment of all the significant cis-sQTL or cis-eQTL SNPs (without SNP selection) for heritability of the twelve brain-related traits. In panel **a**, $\mathrm{Pr}(h_\mathrm{g}^2)$ is a ratio of heritability attributable to all the significant cis-sQTL (or cis-eQTL) SNPs to the overall SNP-based heritability ($h_{\mathrm{xQTL}}^2/h_{\mathrm{SNP}}^2$). In panel **b**, heritability enrichment is defined as a ratio of $\mathrm{Pr}(h_\mathrm{g}^2)$ to the proportion of the SNPs, i.e., $\mathrm{Pr}(h_g^2)\,/\mathrm{Pr}\ (\mathrm{SNPs})$ with $\mathrm{Pr(SNPs)} = \frac{\#\ \text{of SNPs in query}}{\#\ \text{of SNPs in total}}$. The blue dashed line in panel **a** or **b** represents the median value across traits. In panel **c**, the S-LDSC parameter τ is used to assess the contribution of all the significant cis-sQTL (or cis-eQTL) SNPs to heritability when fitted jointly with all the significant cis-eQTL (or cis-sQTL) SNPs. Each column represent a point estimate with an error bar indicating the 95% CI of the estimate.

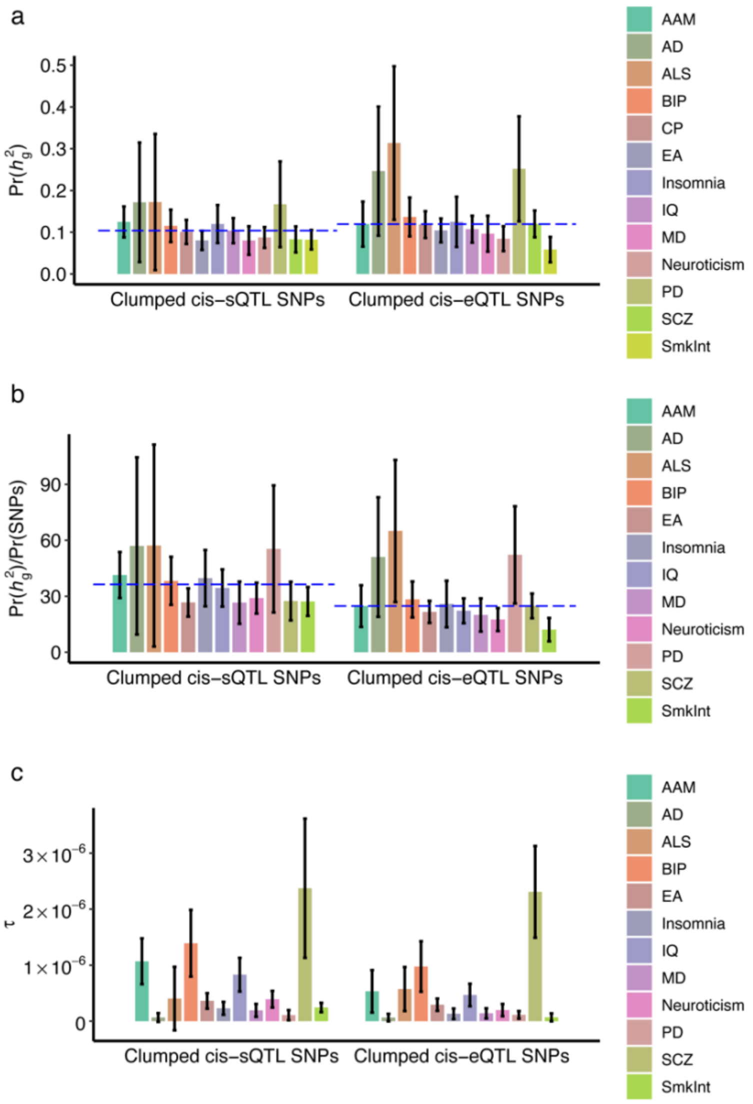

Supplementary Figure 18. Enrichment of the clumped cis-sQTL or cis-eQTL SNPs for heritability of the twelve brain-related traits. To select independently associated cis-sQTL/eQTL SNPs, we performed PLINK clumping analyses with the cis-sQTL or cis-eQTL summary statistics using an LD r^2 threshold of 0.10, a window size of 2 Mb, and a p-value threshold of 5×10^{-8}. In panel **a**, $\Pr(h_g^2)$ is a ratio of heritability attributable to the clumped cis-sQTL or cis-eQTL SNPs to the overall SNP-based heritability ($h_{\text{xQTL}}^2/h_{\text{SNP}}^2$). In panel **b**, heritability enrichment is defined as a ratio of $\Pr(h_g^2)$ to the proportion of the SNPs, i.e., $\Pr(h_g^2)/\Pr(\text{SNPs})$ with $\Pr(\text{SNPs}) = \frac{\text{\# of SNPs in query}}{\text{\# of SNPs in total}}$. The blue dashed line in panel **a** or **b** represents the median value across traits. In panel **c**, the S-LDSC parameter τ is used to assess the contribution of the clumped cis-sQTL (or cis-eQTL) SNPs to heritability when fitted jointly with the clumped cis-eQTL (or cis-sQTL) SNPs. Each column represent a point estimate with an error bar indicating the 95% CI of the estimate.

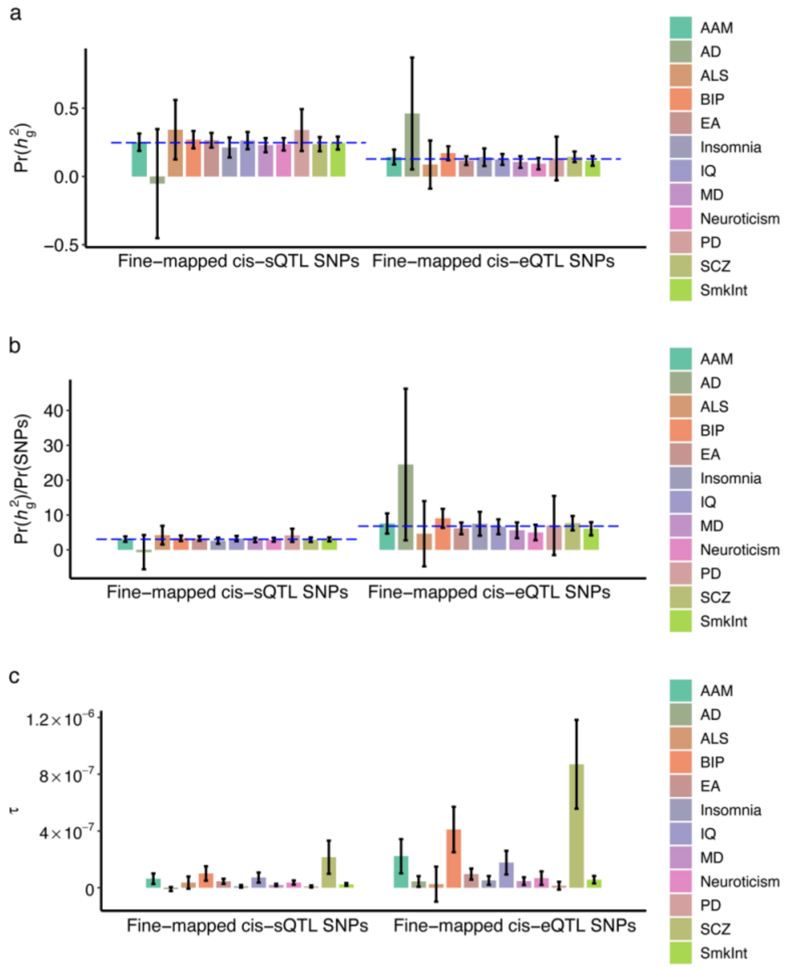

Supplementary Figure 19. Enrichment of the fine-mapped cis-sQTL or cis-eQTL SNPs for heritability of the twelve brain-related traits. We fine-mapped each cis-sQTL (or cis-eQTL) region and computed the causal posterior probability (CPP) of each SNP in the region using SuSiE[23]. Note that only the LeafCutter & QTLtools sQTLs were analyzed with SuSiE because it requires signed z-statistics which are not available for the THISTLE sQTLs. We assigned the maximum CCP across all genes (or introns) to an SNP as its annotation value and a zero value to an SNP that does not belong to any 95% credible set. In panel **a**, $\Pr\left(h_{\mathrm{g}}^{2}\right)$ is a ratio of heritability attributable to the fine-mapped cis-sQTL or cis-eQTL SNPs to the overall SNP-based heritability ($h_{\mathrm{xQTL}}^{2}/h_{\mathrm{SNP}}^{2}$). In panel **b**, heritability enrichment is defined as a ratio of $\Pr\left(h_{\mathrm{g}}^{2}\right)$ to the proportion of the SNPs, i.e., $\Pr\left(h_{g}^{2}\right)/\Pr\,(\mathrm{SNPs})$ with $\Pr(\mathrm{SNPs}) = \frac{\#\text{ of SNPs in query}}{\#\text{ SNPs in total}}$. The blue dashed line in panel **a** or **b** represents the median value across traits. In panel **c**, the S-LDSC parameter τ is used to assess the contribution of the fine-mapped cis-sQTL (or cis-eQTL) SNPs to heritability when fitted jointly with the fine-mapped cis-eQTL (or cis-sQTL) SNPs. Each column represent a point estimate with an error bar indicating the 95% CI of the estimate.

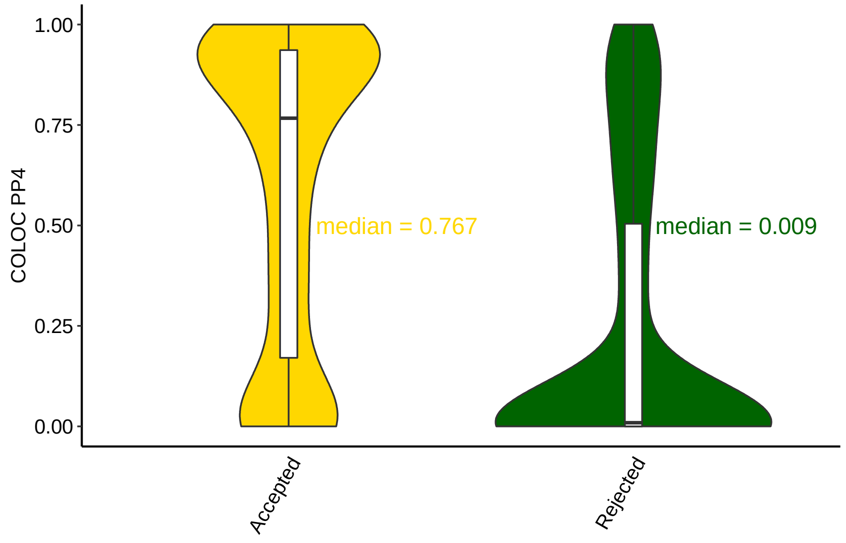

Supplementary Figure 20. COLOC PP4 values of the SMR associations accepted ($P_{\text{HEIDI}} > 0.01$) or rejected ($P_{\text{HEIDI}} < 0.01$) by the HEIDI test. SMR & HEIDI analysis was conducted using the eQTL summary statistics from the whole dataset and GWAS data for the twelve brain-related phenotypes. Shown in the two violin plots are the PP4 values for 443 and 311 SMR associations accepted and rejected by the HEIDI test, respectively. The line inside each box indicates the median value, notches indicate the 95% CI, the central box indicates the IQR, and whiskers indicate data up to 1.5 times the IQR.

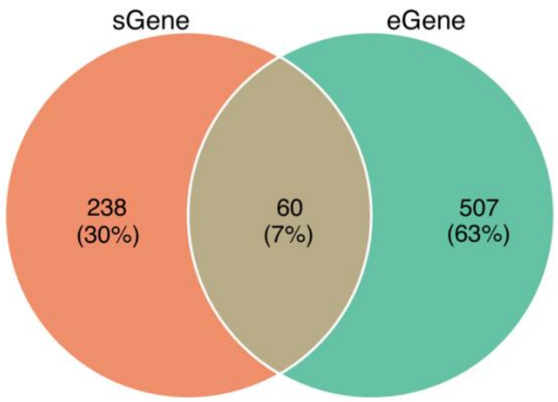

Supplementary Figure 21. Comparison between the trait-associated sGenes and eGenes identified by FOCUS. Only the sQTLs discovered by LeafCutter & QTLtools were included in the FOCUS analysis.

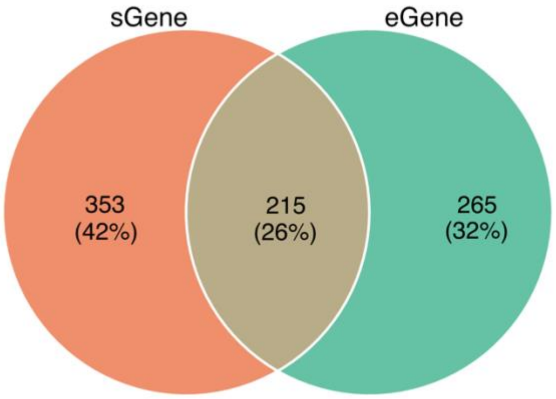

Supplementary Figure 22. Overlap between the trait-associated sGenes and eGenes identified by SMR & COLOC PP4 for 7 additional traits. The 7 traits are from extraordinarily large GWAS (n > 600,000) of height, body mass index (BMI), type 2 diabetes (T2D), low-density lipoprotein cholesterol (LDL), high-density lipoprotein cholesterol (HDL), triglycerides (TG), and total cholesterol (TC).

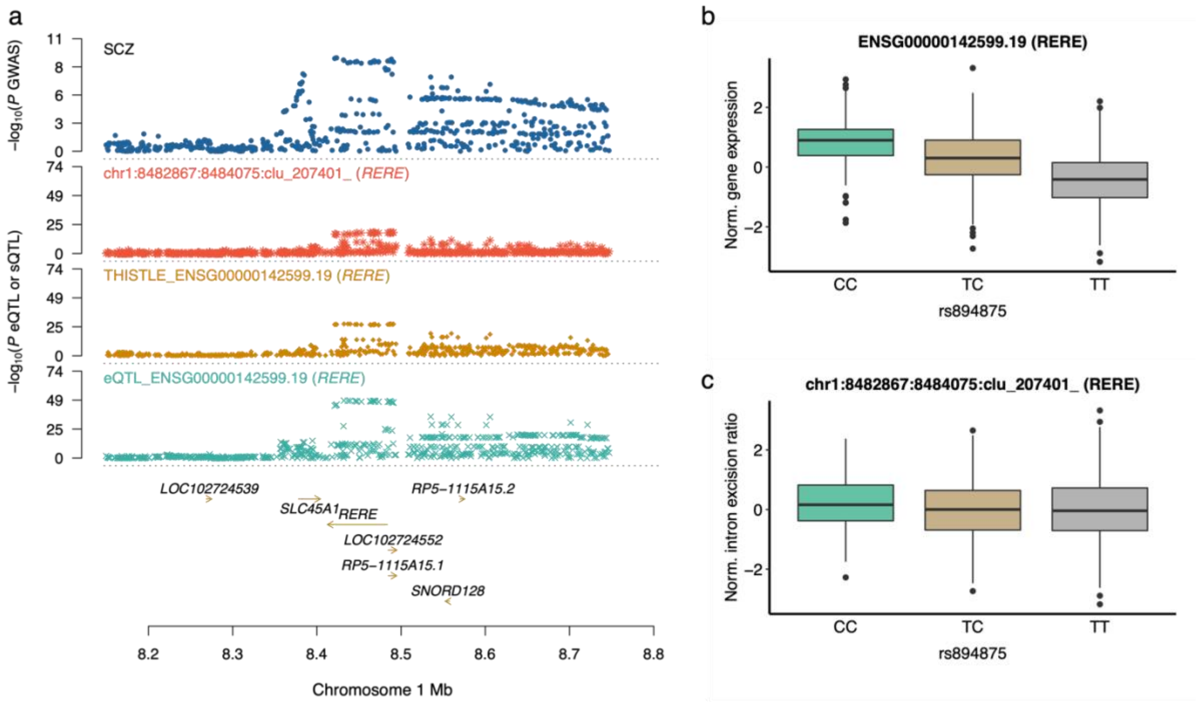

Supplementary Figure 23. Association of *RERE* with schizophrenia through two distinct genetic regulatory mechanisms. **a**) The top track shows −log10(p-values) of SNPs from the schizophrenia GWAS. The second, third, and fourth tracks show −log10(p-values) from the LeafCutter & QTLtools sQTL analysis for intron 1:8482867:8484075:clu_207401_ of *RERE*, THISTLE sQTL analysis for *RERE*, and eQTL analysis for *RERE*, respectively. The THISTLE sQTL p-values were computed using a one-sided sum of chi-squared test, and the eQTL and LeafCutter & QTLtools sQTL p-values were computed using a one-sided chi-squared test. **b**) Association of rs894875 (the lead eQTL SNP) with the overall mRNA abundance of *RERE*. **c**) Association of rs894875 with intron excision ratio of 1:8482867:8484075:clu_207401_. Each boxplot shows the distribution of mRNA abundances (**b**) or intron excision ratios (**c**) in a genotype class, i.e., CC (n = 75), CT (n = 361) or TT (n = 396). The line inside each box indicates the median value, notches indicate the 95% CI, the central box indicates the IQR, whiskers indicate data up to 1.5 times the IQR, and outliers are shown as separate dots.

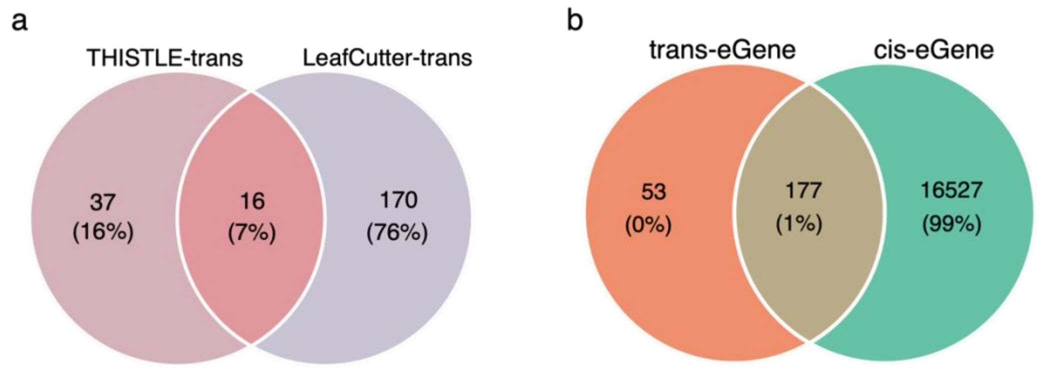

Supplementary Figure 24. Comparison of the trans-sGenes between THISTLE and LeafCutter & QTLtools (**a**) and between the trans-eGenes and cis-eGenes (**b**).

Supplementary Tables

Supplementary Table 1. Number of individuals in each dataset after quality control

Dataset	N	m	Genotyping	Paired-end RNA-seq
LIBD	128	6,650,820	Array	Yes
HBCC	129	6,565,722	Array	Yes
BM10 (MSBB)	183	10,444,061	WGS	No
BM22 (MSBB)	158	10,444,061	WGS	No
BM36 (MSBB)	138	10,444,061	WGS	No
BM44 (MSBB)	156	10,444,061	WGS	Yes
Mayo	288	9,820,326	WGS	Yes
BrainGVEX	384	7,064,078	Array	Yes
CMC	469	7,396,529	Array	Yes
ROSMAP	832	10,301,379	WGS	Yes
Total	2,865 (2,443)	11,645,841	/	/

N: sample size; **m:** number of variants; **WGS**: whole-genome sequencing.

Supplementary Table 2. Summary of cis-sQTLs or cis-eQTLs identified in this study

Method	Variants tested	Genes tested	Sig. associations	xQTL SNPs	sGenes or eGenes
THISTLE	11,631,763	18,559	2,092,808	1,342,073	9,305
LeafCutter & QTLtools	11,631,763	14,085	6,767,649	1,371,483	8,602
Combined sQTL	11,631,763	21,068	8,860,457	1,864,200	12,794
eQTL	11,631,763	31,401	3,765,698	1,962,114	16,704

Supplementary Table 3. Permutation-based p-value threshold to control the overall FDR < 0.05 in each RNA-seq dataset

Dataset	cis-eQTL		THISTLE cis-sQTL		LeafCutter & QTLtools cis-sQTL	
	Permutation -based p-value threshold	Overlap proportion	Permutation -based p-value threshold	Overlap proportion	Permutation -based p-value threshold	Overlap proportion
LIBD	5.0e-06	50.5%	1.2e-05	46.6%	3.1e-07	72.4%
HBCC	6.4e-06	56.9%	4.1e-05	40.6%	2.0e-07	86.0%
BM10	1.5e-06	67.9%	1.3e-06	70.3%	7.4e-08	94.0%
BM22	3.4e-06	54.6%	1.2e-06	67.2%	1.7e-07	77.3%
BM36	1.9e-06	58.0%	1.3e-06	62.5%	2.1e-07	67.7%
BM44	3.2e-06	57.7%	2.4e-06	59.3%	5.5e-08	99.2%
Mayo	1.5e-05	58.5%	1.5e-05	65.6%	7.9e-07	85.6%
BrainGVEx	2.8e-05	56.3%	1.9e-05	61.4%	1.8e-07	90.7%
CMC	3.0e-05	59.3%	8.4e-06	70.6%	1.9e-07	91.4%
ROSMAP	3.4e-05	69.4%	3.3e-05	69.4%	5.9e-07	83.7%

Permutation-based p-value threshold: a p-value threshold computed from a permutation-based procedure to control an overall false-discovery rate (FDR) at 0.05. **Overlap proportion:** the proportion of eGenes or sGenes identified with p < 5e-08 overlapped with that identified with FDR < 0.05. The THISTLE sQTL p-values were computed using a one-sided sum of chi-squared test, and the eQTL and LeafCutter & QTLtools sQTL p-values were computed using a one-sided chi-squared test.

Supplementary Table 4. Comparison of sQTLs between this study and the previous studies

Study	Method	Sample size	No. of sGenes	No. of sQTLs
Guelfi et al.[16]	Altrans & MatrixEQTL	117	\	\
Takata et al.[17]	Vast-tools & MatrixEQTL	206	1,341	8,966
Raj et al.[24]	LeafCutter & FastQTL	450	3,006	9,028
Wang et al.[19] (with filtering)	QTLtools	1,387	2,064	196,930
Wang et al.[19] (no filtering)	QTLtools	1,387	7,296	462,722
This study (with filtering)	THISTL, LeafCutter & QTLtools	2,865	12,794	1,864,200
This study (no filtering)	THISTLE, LeafCutter & QTLtools	2,865	13,716	1,875,864

There are two versions of the Wang et al. sQTL summary statistics available, with one version excluding transcripts whose expression levels were < 5 FPKM in all the individuals and the other version without such filtering. In our study, there were also two versions of sQTL summary statistics, with one version excluding transcripts whose expression levels were < 0.1 TPM in more than 80% of individuals and the other version without such filtering. The Guelfi et al.[16], Takata et al.[17], and Raj et al.[24] studies did not release the full sQTL summary statistics. Takata et al. and Raj et al. reported the number of sQTLs and sGenes at 5% FDR. For our data and the Wang et al. data, we reported the number of sQTLs and sGenes at $P_{\text{sQTL}} < 5 \times 10^{-8}$.

Supplementary Table 5. GWAS summary data

Complex traits	n	n_{case}	$n_{control}$	No. of SNPs
Educational attainment (EA)	766,345	/	/	10,101,242
Smoking initiation (SmkInt)	632,802	311,629	321,173	7,731,760
Major depression (MD)	500,199	170,756	329,443	8,483,302
Parkinson's disease (PD)	482,730	33,674	449,056	17,481,233
Neuroticism	449,484	/	/	7,374,623
Alzheimer's disease (AD)	387,000	71,880	315,120	13,283,327
Insomnia	386,533	109,402	277,131	10,862,567
Age at menarche (AAM)	370,000	/	/	8,525,686
Schizophrenia (SCZ)	306,011	69,369	236,642	5,426,250
Intelligence (IQ)	269,867	/	/	9,295,118
Amyotrophic lateral sclerosis (ALS)	138,086	27,205	110,881	8,709,431
Bipolar disorder (BIP)	51,710	41,917	371,549	13,413,244

n: sample size; n_{case}: number of cases; $n_{control}$: number of controls. IQ was assessed using various neurocognitive tests, primarily gauging fluid domains of cognitive functioning[25]. EA was measured as the number of years of schooling that individuals completed[26]. AAM is a female-specific trait, referring to the age when periods started. Neuroticism was measured with 12 dichotomous items of the Eysenck Personality Questionnaire Revised Short Form[27].

Supplementary Table 6. Number of genes associated with the twelve brain-related phenotypes identified by SMR & COLOC PP4.

Trait	LeafCutter & QTLtools sQTLs			THISTLE sQTLs			eQTLs		
	No. of introns (genes) tested	No. of introns (genes) after SMR	No. of introns (genes) after COLOC PP4	No. of genes tested	No. of genes after SMR	No. of genes after COLOC PP4	No. of genes tested	No. of genes after SMR	No. of genes after COLOC PP4
EA	45,481(7,760)	315(90)	114 (33)	8,611	167	53	15,350	216	59
SmkInt	43,408(7,609)	42(14)	15(8)	8,733	25	12	15,443	35	21
MD	46,152(7,815)	38(7)	33(6)	8,675	6	5	15,478	15	13
PD	46,227(7,839)	76(15)	11(4)	8,686	21	3	15,516	41	10
Neuroticism	43,862(7,651)	80(14)	9(5)	8,759	40	13	15,506	52	17
AD	43,987(7,666)	34(14)	5(2)	8,765	18	4	15,529	20	3
Insomnia	46,177(7,837)	29(1)	0(0)	8,682	4	2	15,501	6	2
AAM	44,262(7,690)	106(49)	36(18)	8,792	94	24	15,574	124	31
SCZ	42,794(7,499)	158(66)	41(19)	8,661	120	41	15,242	139	45
IQ	46,121(7,825)	128(42)	50(21)	8,679	99	30	15,493	116	31
BIP	43,209(7,572)	44(20)	21(13)	8,704	29	17	15,331	33	18
ALS	42,367 (7,470)	8(3)	3(2)	8,613	8	3	15,153	8	5
Total	/	796 (255)	317 (114)	/	473	184	/	577	226

Genes (introns) associated with the brain-related traits were identified by an analysis (SMR & COLOC PP4) that integrates the LeafCutter & QTLtools sQTLs, THISTLE sQTLs, or eQTLs into GWAS. The twelve brain-related traits are intelligence (IQ), educational attainment (EA), smoking initiation (SmkInt), schizophrenia (SCZ), Alzheimer's disease (AD), insomnia, bipolar disorder (BIP), major depression (MD), Parkinson's disease (PD), neuroticism, age at menarche (AAM), and amyotrophic lateral sclerosis (ALS). **No. of introns (gene) tested:** the number of introns included in the SMR analysis with the number of unique genes shown in the parentheses. **No. of genes tested**: the number of genes included in the SMR analysis. **No. of introns (genes) after SMR**: the number of introns associated with a phenotype at a genome-wide significance level in the SMR analysis with the number of unique genes shown in the parentheses. **No. of genes after SMR**: the number of genes associated with a phenotype at a genome-wide significance level in the SMR analysis. **No. of genes after COLOC PP4**: the number of genes passed the SMR test and showed a COLOC PP4 value of > 0.8.

Supplementary Table 7. Number of genes associated with the twelve brain-related phenotypes identified by FOCUS

Trait	LeafCutter & QTLtools sQTL		eQTLs	
	**No. of genes tested**	**No. of sig. genes (FUSION/FOCUS)**	**No. of genes tested**	**No. of sig. genes (FUSION/FOCUS)**
EA	3968	253/87	6,624	441/158
SmkInt	742	42/13	1,633	108/49
MD	524	22/8	848	61/15
PD	369	45/18	592	74/28
Neuroticism	994	78/22	1,639	132/39
AD	616	29/8	962	54/11
Insomnia	173	11/2	256	27/4
AAM	2,415	165/58	3,850	288/101
SCZ	2,466	132/49	4,418	338/115
IQ	2,422	161/59	3,897	284/93
BIP	878	45/16	1,408	88/24
ALS	174	8/0	275	10/2
Total	/	754/298	/	1,372/567

Genes associated with the brain-related traits were identified by an analysis with FOCUS that integrates the LeafCutter & QTLtools sQTL or eQTL data into GWAS. **No. of genes tested**: the number of genes included in the FOCUS analysis. **No. of sig. genes (FUSION/FOCUS)**: the number of genes associated with a phenotype identified by FUSION[28] (the initial step of FOCUS) or FOCUS at a genome-wide significance level.

Supplementary References

1. Lappalainen, T. *et al.* Transcriptome and genome sequencing uncovers functional variation in humans. *Nature* **501**, 506-511 (2013).
2. Battle, A. *et al.* Characterizing the genetic basis of transcriptome diversity through RNA-sequencing of 922 individuals. *Genome Research* **24**, 14-24 (2014).
3. Monlong, J., Calvo, M., Ferreira, P.G. & Guigó, R. Identification of genetic variants associated with alternative splicing using sQTLseekeR. *Nature Communications* **5**, 4698 (2014).
4. Shen, S. *et al.* rMATS: robust and flexible detection of differential alternative splicing from replicate RNA-Seq data. *Proceedings of the National Academy of Sciences* **111**, E5593-E5601 (2014).
5. Ongen, H. & Dermitzakis, E.T. Alternative splicing QTLs in European and African populations. *The American Journal of Human Genetics* **97**, 567-575 (2015).
6. Vaquero-Garcia, J. *et al.* A new view of transcriptome complexity and regulation through the lens of local splicing variations. *eLife* **5**, e11752 (2016).
7. Li, Y.I. *et al.* Annotation-free quantification of RNA splicing using LeafCutter. *Nature Genetics* **50**, 151 (2018).
8. Frazee, A.C., Jaffe, A.E., Langmead, B. & Leek, J.T. Polyester: simulating RNA-seq datasets with differential transcript expression. *Bioinformatics* **31**, 2778-2784 (2015).
9. Garrido-Martín, D., Borsari, B., Calvo, M., Reverter, F. & Guigó, R. Identification and analysis of splicing quantitative trait loci across multiple tissues in the human genome. *Nature Communications* **12**, 1-16 (2021).
10. Dobin, A. *et al.* STAR: ultrafast universal RNA-seq aligner. *Bioinformatics* **29**, 15-21 (2013).
11. Li, B. & Dewey, C.N. RSEM: accurate transcript quantification from RNA-Seq data with or without a reference genome. *BMC Bioinformatics* **12**, 323 (2011).
12. Nowicka, M. & Robinson, M.D. DRIMSeq: a Dirichlet-multinomial framework for multivariate count outcomes in genomics. *F1000Research* **5**(2016).
13. 1000 Genomes Project Consortium. An integrated map of genetic variation from 1,092 human genomes. *Nature* **491**, 56 (2012).
14. International HapMap 3 Consortium. Integrating common and rare genetic variation in diverse human populations. *Nature* **467**, 52 (2010).
15. Storey, J.D. & Tibshirani, R. Statistical significance for genomewide studies. *Proceedings of the National Academy of Sciences* **100**, 9440-9445 (2003).

16. Guelfi, S. *et al.* Regulatory sites for splicing in human basal ganglia are enriched for disease-relevant information. *Nature Communications* **11**, 1-16 (2020).
17. Takata, A., Matsumoto, N. & Kato, T. Genome-wide identification of splicing QTLs in the human brain and their enrichment among schizophrenia-associated loci. *Nature Communications* **8**, 1-11 (2017).
18. Li, Y.I., Wong, G., Humphrey, J. & Raj, T. Prioritizing Parkinson's disease genes using population-scale transcriptomic data. *Nature Communications* **10**, 1-10 (2019).
19. Wang, D. *et al.* Comprehensive functional genomic resource and integrative model for the human brain. *Science* **362**(2018).
20. Lynch, M. & Walsh, B. *Genetics and analysis of quantitative traits*, (Sinauer Sunderland, MA, 1998).
21. Wenger, A.M. *et al.* Accurate circular consensus long-read sequencing improves variant detection and assembly of a human genome. *Nature Biotechnology* **37**, 1155-1162 (2019).
22. Hoffman, G.E. & Schadt, E.E. variancePartition: interpreting drivers of variation in complex gene expression studies. *BMC Bioinformatics* **17**, 483 (2016).
23. Wang, G., Sarkar, A., Carbonetto, P. & Stephens, M. A simple new approach to variable selection in regression, with application to genetic fine mapping. *Journal of the Royal Statistical Society: Series B (Statistical Methodology)* **82**, 1273-1300 (2020).
24. Raj, T. *et al.* Integrative transcriptome analyses of the aging brain implicate altered splicing in Alzheimer's disease susceptibility. *Nature Genetics* **50**, 1584 (2018).
25. Savage, J.E. *et al.* Genome-wide association meta-analysis in 269,867 individuals identifies new genetic and functional links to intelligence. *Nature genetics* **50**, 912 (2018).
26. Lee, J.J. *et al.* Gene discovery and polygenic prediction from a genome-wide association study of educational attainment in 1.1 million individuals. *Nature Genetics* **50**, 1112 (2018).
27. Eysenck, S.B., Eysenck, H.J. & Barrett, P. A revised version of the psychoticism scale. *Personality and Individual Differences* **6**, 21-29 (1985).
28. Gusev, A. *et al.* Integrative approaches for large-scale transcriptome-wide association studies. *Nature Genetics* **48**, 245-252 (2016).
