# Supplementary material for: Genetic control of RNA splicing and its distinct role in complex trait variation
Source: Nat Genet. 2022 Aug 18;54(9):1355–63. doi: 10.1038/s41588-022-01154-4 (PMC9470536; doi:10.1038/s41588-022-01154-4)
Supplement: Supplementary file 2 — Reporting Summary [file 41588_2022_1154_MOESM2_ESM.pdf]

nature research

Corresponding author(s): Jian Yang

Last updated by author(s): Jun 6, 2022

Reporting Summary

Nature Research wishes to improve the reproducibility of the work that we publish. This form provides structure for consistency and transparency in reporting. For further information on Nature Research policies, see our Editorial Policies and the Editorial Policy Checklist.

Statistics

For all statistical analyses, confirm that the following items are present in the figure legend, table legend, main text, or Methods section.

n/a	Confirmed	
☐	☒	The exact sample size (*n*) for each experimental group/condition, given as a discrete number and unit of measurement
☒	☐	A statement on whether measurements were taken from distinct samples or whether the same sample was measured repeatedly
☐	☒	The statistical test(s) used AND whether they are one- or two-sided<br>*Only common tests should be described solely by name; describe more complex techniques in the Methods section.*
☐	☒	A description of all covariates tested
☐	☒	A description of any assumptions or corrections, such as tests of normality and adjustment for multiple comparisons
☐	☒	A full description of the statistical parameters including central tendency (e.g. means) or other basic estimates (e.g. regression coefficient) AND variation (e.g. standard deviation) or associated estimates of uncertainty (e.g. confidence intervals)
☐	☒	For null hypothesis testing, the test statistic (e.g. *F*, *t*, *r*) with confidence intervals, effect sizes, degrees of freedom and *P* value noted<br>*Give P values as exact values whenever suitable.*
☒	☐	For Bayesian analysis, information on the choice of priors and Markov chain Monte Carlo settings
☒	☐	For hierarchical and complex designs, identification of the appropriate level for tests and full reporting of outcomes
☐	☒	Estimates of effect sizes (e.g. Cohen's *d*, Pearson's *r*), indicating how they were calculated

Our web collection on statistics for biologists contains articles on many of the points above.

Software and code

Policy information about availability of computer code

Data collection	We analyzed existing datasets. Thus, no software was used to collect data.
Data analysis	STAR v2.7.8a (https://github.com/alexdobin/STAR) was used for RNA-seq reads alignment. RNA-SeQC v2.3.5 (https://github.com/getzlab/rnaseqc) was used for quantifying gene-level abundance. RSEM v1.3.1 (https://github.com/deweylab/RSEM) was used for quantifying isoform-level abundance. OSCA v0.45 (https://yanglab.westlake.edu.cn/software/osca; https://github.com/jianyangqt/osca) was used for the THISTLE sQTL analysis. LeafCutter v0.2.9 (https://davidaknowles.github.io/leafcutter/articles/sQTL.html) and fastQTL v2 .184 (https://http://fastqtl.sourceforge.net/) were used for the LeafCutter sQTL analysis. S-LDSC v1.0.1 (https://github.com/bulik/ldsc) was used for the heritability enrichment analysis. MESC v1 (https://github.com/douglasyao/mesc) was used for the mediation heritability analysis. SMR v1.03 (https://yanglab.westlake.edu.cn/software/smr/#Overview) was used for the SMR analysis. COLOC v5.1.1 (https://cran.r-project.org/web/packages/coloc/index.html) and eCAVIAR v2.2 (https://github.com/fhormoz/caviar) were used for the COLOC analysis. TORUS v1 (https://github.com/xqwen/torus) was used for the functional enrichment analysis. FOCUS v0.6.10 (https://github.com/bogdanlab/focus) was used for fine-mapping causal genes.

For manuscripts utilizing custom algorithms or software that are central to the research but not yet described in published literature, software must be made available to editors and reviewers. We strongly encourage code deposition in a community repository (e.g. GitHub). See the Nature Research guidelines for submitting code & software for further information.

Data

Policy information about availability of data

All manuscripts must include a data availability statement. This statement should provide the following information, where applicable:

- Accession codes, unique identifiers, or web links for publicly available datasets
- A list of figures that have associated raw data
- A description of any restrictions on data availability

PsychENCODE data: https://www.synapse.org/#!Synapse:syn4921369/files/. AMP-AD data: https://www.synapse.org/#!Synapse:syn5550382. Online tool for querying the sQTL and eQTL summary statistics: https://yanglab.westlake.edu.cn/data/brainmeta. The full summary statistics from the sQTL, eQTL, SMR and COLOC analyses are available at https://yanglab.westlake.edu.cn/pub_data.html. GRCh37 genome: https://www.ncbi.nlm.nih.gov/genome/guide/human/. GENCODE: https://www.gencodegenes.org/human/release_37lift37.html.

Field-specific reporting

Please select the one below that is the best fit for your research. If you are not sure, read the appropriate sections before making your selection.

☒ Life sciences ☐ Behavioural & social sciences ☐ Ecological, evolutionary & environmental sciences

For a reference copy of the document with all sections, see nature.com/documents/nr-reporting-summary-flat.pdf

Life sciences study design

All studies must disclose on these points even when the disclosure is negative.

Sample size	We used 2,443 unrelated individuals of European ancestry from the Psych ENCODE and AMP-AD consortia for real data analysis. The sample size was determined by the maximum number of unrelated individuals of European ancestry with both SNP genotype and RNA-seq data in the PsychENCODE and AMP-AD consortia.
Data exclusions	We excluded individuals of non-European ancestry, with < 10 million total reads, or with RNA integrity number < 5.5, and one of each pair of individuals with genetic relatedness > 0.05. We excluded genetic variants with minor allele frequencies < 0.01, Hardy- Weinberg Equilibrium test P-value < le-6, imputation INFO score < 0.3, or missingness rate > 0.05.
Replication	We replicated the simulation 500 times in each scenario to investigate the performance of THISTLE in comparison with existing methods. All replications were successfully performed. In real data analysis, we used all the available data to maximize power for discovery, so replication was not performed.
Randomization	We analyzed existing data sets. Thus, no randomization was performed. In eQTL and sQTL analysis, covariates were adjusted for to account for potential confounding.
Blinding	Group allocation was not relevant to this study, so blinding was not necessary.

Reporting for specific materials, systems and methods

We require information from authors about some types of materials, experimental systems and methods used in many studies. Here, indicate whether each material, system or method listed is relevant to your study. If you are not sure if a list item applies to your research, read the appropriate section before selecting a response.

Materials & experimental systems

n/a	Involved in the study	
☒	☐	Antibodies
☒	☐	Eukaryotic cell lines
☒	☐	Palaeontology and archaeology
☒	☐	Animals and other organisms
☐	☒	Human research participants
☒	☐	Clinical data
☒	☐	Dual use research of concern

Methods

n/a	Involved in the study	
☒	☐	ChIP-seq
☒	☐	Flow cytometry
☒	☐	MRI-based neuroimaging

Human research participants

Policy information about studies involving human research participants

Population characteristics	Our study involved publicly available data sets (e.g. PsychENCODE, AMDAD, and existing summary statistics). We restricted our data to individuals of European ancestry.

Recruitment	We analyzed existing data sets. Thus, no recruitment was performed.
Ethics oversight	The Ethics Committee of Westlake University (approval number: 20200722YJ001) and the University of Queensland Human Research Ethics Committee B (approval number: 2011001173).

Note that full information on the approval of the study protocol must also be provided in the manuscript.
